# Supplementary material for: Aquificae overcomes competition by archaeal thermophiles, and crowding by bacterial mesophiles, to dominate the boiling vent-water of a Trans-Himalayan sulfur-borax spring
Source: PLoS One. 2024 Oct 25;19(10):e0310595. doi: 10.1371/journal.pone.0310595 (PMC11508158; doi:10.1371/journal.pone.0310595)
Supplement: S1 File — (DOC) [file pone.0310595.s001.doc]

**Supplementary Information**

**Aquificae overcomes competition by archaeal thermophiles, and crowding by bacterial mesophiles, to dominate the boiling vent-water of a Trans-Himalayan sulfur-borax spring**

Nibendu Mondal^1,$^, Subhajit Dutta^1^, Sumit Chatterjee^1^, Jagannath Sarkar^1^, Mahamadul Mondal^1^, Chayan Roy^2^, Ranadhir Chakraborty^3^ and Wriddhiman Ghosh^1^,*

**Author Addresses:**

^1^ Department of Biological Sciences, Bose Institute, Kolkata, India

^2^ Department of Plant and Environmental Sciences, University of Copenhagen, Copenhagen, Denmark

^3^ Department of Biotechnology, University of North Bengal, Siliguri, India

**Present Address:**

^$^ International Institute of Innovation and Technology, Kolkata, India

*** Correspondence:** Wriddhiman Ghosh (wriman@jcbose.ac.in / Wriman@rediffmail.com)

**Keywords:** Trans-Himalayan sulfur-borax hot springs, vent-water microbiome, hot spring mesophiles, Proteobacteria, Aquificae

**Table of Contents**

**Supplementary Method**

- Enumeration of microbial cells

**Supplementary Results**

- Taxonomic relationships of the population genome bins obtained from the assembled metagenome
- Metabolically specialized (environmentally advantageous) functions encoded by the Lotus Pond MAGs
- Metagenome-encoded functions that can help bacterial mesophiles survive in the Lotus Pond habitat

**Supplementary Tables**

**Table S1.** Summary of the metagenomic sequence dataset generated for Lotus Pond’s vent-water.

**Table S2.** Complete annotation of the 220687 protein coding sequences identified within the assembled metagenome of Lotus Pond's vent-water by searching against the eggNOG database v5.0 using eggNOG-mapper v2.1.9. Since this Table is more than one page long it has been provided as an Excel sheet named Table S2, within the Excel Workbook named Supplementary_Dataset.

**Table S3.** Taxonomic affiliation of the archaeal and bacterial protein-coding sequences predicted within the assembled metagenome of Lotus Pond’s vent-water.

**Table S4.** Taxonomic classification of the metagenomic reads which corresponded to archaeal and bacterial 16S rRNA gene sequences.

**Table S5.** Taxonomic classification of the OTUs obtained via PCR-amplified 16S rRNA gene sequence analysis.

**Table S6.** Characteristic features of the population genome bins constructed from the assembled metagenome of Lotus Pond's vent-water. Since this Table is more than one page long it has been provided as an Excel sheet named Table S6, within the Excel Workbook named Supplementary_Dataset.

**Table S7.** Metabolic categories over which Lotus Pond's assembled-metagenomic ORFs ascribable to clusters of orthologous genes (COGs) were distributed (genes were identified as belonging to COGs by searching against the COG Letter edition database of NCBI using COGclassifier v1.0.5). Since this Table is more than one page long it has been provided as an Excel sheet named Table S7, within the Excel Workbook named Supplementary_Dataset.

**Table S8.** Taxonomic distribution of all eggNOG-annotated CDSs of the Lotus Pond metagenome that were directly or indirectly related to the biosynthesis or resistance of secondary metabolites and antibiotics.

**Supplementary References**

- References used in Supplementary Methods and Supplementary Results
- References used in Tables S4, S5 and S6

**Supplementary Figure**

**Fig S1.** Rarefaction curves showing the proportionality between OTU-level diversity revealed and the number of 16S rRNA gene sequence reads analyzed, for archaea or bacteria, in the vent-water sample.

**Supplementary Method**

**Enumeration of microbial cells**

Total microbial cell count per unit volume of Lotus Pond’s vent-water was determined in tandem with the number of viable and non-viable cells present. The microbial cells that were filtered out from 500 mL vent-water in a 0.22 μm mixed cellulose ester membrane filter were first detached from the adherence matrix by shredding the filter with sterile scissors, and then vortexing for 30 min, within the NaCl-glycerol containing cryovial in which the filter was inserted on field. The vial was spun at 1000 *g* for 5 seconds to allow the filter shreds to settle at the bottom of the container. Subsequently, the supernatant was collected (without disturbing the bottom debris), its exact volume was measured, and the suspension was subjected to microscopic analyses with the axiom that it contained all the microbial cells that were present in the 500 mL vent-water, which was passed through the 0.22 μm filter, during the field expedition on 29 October 2021.

From the cell suspension curated above, 50 μL was stained by mixing 4 μL of a 4′6-diamidino-2-phenylindole (DAPI) solution that had a concentration of 10 µg mL^-1^; the mixture was kept in the dark, at 40°C, for 15 minutes (Dong et al., 2016). Another 50 μL fraction of the cell suspension was dually stained by mixing 2 μL each of a fluorescein diacetate (FDA) solution and a propidium iodide (PI) solution having a concentration of 25 mg mL^-1^ and 5 mg mL^-1^ respectively (Jones and Senft, 1985). The mixture was kept in the dark, at 37°C, for 15 minutes.

Post incubation, both the types of stained cells were washed twice with sterile phosphate buffered saline (PBS) solution and re-suspended in 50 µL PBS individually. Finally, 20 µL of a stained and washed cell suspension was dispensed to a hemocytometer (Paul Marienfeld GmbH & Co.KG, Germany) and observed under an upright fluorescence microscope (Olympus BX53 Digital, Olympus Corporation, Japan), as described previously (Quan et al., 2015).

Cell numbers were counted based on hemocytometer principles laid down by Absher (1973). For this purpose such a haemocytometer was taken which encompassed nine 1 mm × 1 mm major squares, of which only the four corner squares were subdivided into 4 × 4 grids each. Each smallest square formed by the grids within a major square had a length of 0.25 mm and a breadth of 0.25 mm. The height of the chamber formed after putting the cover slip in was 0.1 mm. Thus, the volume of cell suspension held within the smallest chamber was 0.25 mm × 0.25 mm × 0.1 mm (i.e. 0.00625 mm^3^). Since each major square encompassed 16 smallest chambers, and there were four gridded major squares in all, stained and washed cell suspension equivalent to 64 × 0.00625 mm^3^ (i.e. 0.4 mm^3^) was harboured within the four gridded major squares after flooding the slide and placing the cover slip. Counting cells in each of the 64 smallest chambers of the hemocytometer, and then summing up, gave the total cell count for 0.4 mm^3^ of the stained and washed suspension. From this number, the cell count mL^-1^ of the suspension, and then mL^-1^ of the vent-water, was calculated step-wise via multiplication of the corresponding dilution factors involved.

**Supplementary Results**

**Taxonomic relationships of the population genome bins obtained from the assembled metagenome**

When all the >2500 nucleotide contigs present in the assembled metagenome were binned using Metabat2 , MaxBin2 , and CONCOCT via separate *in silico* experiments, 19, 14 and 30 metagenome-assembled genomes (MAGs) were obtained respectively. Refinement and optimization of all these bins using DASTool yielded 14 bacterial (Tables 1 and S6; Figure 3) and one archaeal (Tables 1 and S6) MAG for subsequent consideration and analysis: each of these draft genomes had a contamination level of <5%, except the one designated as LotusPond_MAG_Paracoccus_sp._1. Likewise, most of the short-listed MAGs (9 out of 15) had >90% completeness; two, identified as Paracoccus_sp._2 and Unclassified_Thermodesulfobacteriaceae, had <50% completeness; and four, identified as Paracoccus_sp._1, Fervidobacterium_sp., Thermus_sp. and Unclassified_Bacteria, possessed approximately 51.2%, 70.2%, 70.3% and 81.9% completeness respectively. In terms of their total consensus sequence length, the population genomes classified as *Vibrio* *metschnikovii* and *Halomonas* sp. were the largest (approximately 3.5 and 3.4 mb respectively) whereas the one classified as a novel member of the family Thermodesulfobacteriaceae was the smallest (approximately 0.4 mb only). Furthermore, when the 31342225 high quality read-pairs used for metagenome assembly were searched against the 15 population genomes, a sum total of 68.08% read-pairs mapped concordantly on to the MAGs as a whole. Individually, the 15 MAGs accounted for 0.06% to 55.9% of the metagenomic read-pairs analyzed.

The genome bin LotusPond_MAG_Unclassified_Aquificaceae accounted for an overwhelming proportion of the metagenome (55.9% of all read-pairs assembled mapped back to this MAG), while another Aquificales bin identified as *Sulfurihydrogenibium azorense*, a member of Hydrogenothermaceae, accounted for 3.5% of all read-pairs. Four more bins individually represented >1% of the metagenome; these were identifed as (i) an unknown species of the genus *Halomonas*, (ii) an unclassified member of the order Desulfurococcales within the archaeal class Thermoprotei, (iii) the gammaproteobacteium *Vibrio* *metschnikovii*, and (iv) an unknown species of the genus *Thermus*. These MAGs accounted for 2.7%, 1.6%, 1.5% and 1.0% of all metagenomic read-pairs respectively. Seven Lotus Pond MAGs, ascribed to *Tepidimonas* *taiwanensis*, *Tepidimonas* sp., *Fervidobacterium* sp., *Thermosynechococcus* sp., *Paracoccus* sp., an unclassified member of the gammaproteobacterial order Chromatiales, and an unclassified taxon of Bacteria, individually accounted for >0.1% but <1% of the metagenome. The remaining two Lotus Pond MAGs individually represented <0.1% of the metagenome; these were identified as one unclassified member each from the family Thermodesulfobacteriaceae of the monotypic phylum Thermodesulfobacteria, and the genus *Paracoccus*.

Detailed phylogenetic and genomic relationships of the LotusPond MAGs, which led to their taxonomic identification and nomencature are described below.

**LotusPond_MAG_Unclassified_Aquificaceae**, in default TYGS search (where the search engine itself finds closely related strains), exhibited highest genome-genome relatedness with a number of species-level entities belonging to the Aquificaceae genera *Hydrogenobacter* and *Thermocrinis* (13.5% and 13.4% dDDH respectively). Notably, the consensus sequence of this MAG encompassed one 5S, but no 16S or 23S rRNA gene, even though the analysis of Lotus Pond’s metagenomic sequence dataset using Bowtie2 (against the RDP rrnDB database version 5.8) and RDP Classifier in tandem identified 44547 raw reads as parts of 16S rRNA genes of *Hydrogenobacter* species. Nevertheless, the 107 nucleotide sequence of the 5S rRNA gene of this MAG, however, exhibited 100% similarity (at 100% query coverage) with the 5S rRNA gene of *Hydrogenobacter thermophilus*, while similarities with all other Aquificaceae homologs were lower. Notably, the length of the consensus sequence (1489711 nucleotides), and the G+C content (43.32%), of LotusPond_MAG_Unclassified_Aquificaceae was most comparable with the corresponding numbers of the unclassified Aquificaceae isolate UBA11096 (1317975 nucleotides, and 43.3%); considerable similarities were also observed with the median genome lengths and median G+C contents of *Hydrogenobacter* *hydrogenophilus* (1.60 mb and 41.5%), *Hydrogenobacter* *thermophilus* (1.74 mb and 44%) and other *Hydrogenobacter* species (1.31 mb and 41.5%); *Thermocrinis ruber* (1.52 mb and 45.2%) and other *Thermocrinis* members (1.28 mb and 45.3%); and *Aquifex* *aeolicus* (1.30 mb and 42.8%) and other *Aquifex* species (1.01 mb, and 42.6%). Corroborative to the above data, GGDC-based analysis showed that LotusPond_MAG_Unclassified_Aquificaceae possessed 78.30% dDDH with UBA11096.

**LotusPond_MAG_Sulfurihydrogenibium_azorense**, in default TYGS search, exhibited highest genome-genome relatedness with strains of *Sulfurihydrogenibium azorense* (91.2% dDDH) followed by those of *Sulfurihydrogenibium subterraneum* (75.9% dDDH). Its consensus sequence encompassed two 5S, but no 16S or 23S, rRNA genes. Both the 5S rRNA genes were 112-nucleotide long but possessed 97.3% mutual sequence similarity (three substitutions across the 112 nucleotide alignment). Concurrently, one 5S rRNA gene of LotusPond_MAG_Sulfurihydrogenibium_azorense showed 100% sequence similarity (at 100% query coverage) with both the 5S rRNA gene homologs of *Sulfurihydrogenibium azorense* strain Az-Fu1, which in turn had 100% sequence similarity between them; the other 5S homolog of LotusPond_MAG_Sulfurihydrogenibium_azorense exhibited 97.3% sequence similarity with the two Az-Fu1 homologs. The length of the consensus sequence (1516769 nucleotides), and the G+C content (32.77%), of LotusPond_MAG_Sulfurihydrogenibium_azorense was comparable with the median genome lengths and median G+C contents of *S*. *azorense* (1.17 mb, and 33.7%), *S*. *subterraneum* (1.61 mb, and 32.3%), and other *Sulfurihydrogenibium* species (1.58 mb, and 32.4%).

**LotusPond_MAG_Halomonas_sp.**, in default TYGS search, exhibited highest genome-genome relatedness with strains of *Halomonas johnsoniae* (65.9% dDDH), *Halomonas stevensii* (65.3% dDDH) and *Halomonas hamiltonii* (64.7% dDDH), genomic relatedness with other Halomonas species ranged between 21.1% and 46.1% dDDH. The MAG contained six 5S, but no 16S or 23S, rRNA genes. While pair-wise sequence similarity between the 5S rRNA genes ranged between 94.6% and 99.1%, each of the six 111-nucleotide long sequences individually exhibited highest (96.4% to 99.1%) sequence similarity (at 100% query coverage) with homologs from Halomonas axialensis, Halomonas hydrothermalis and/or Halomonas piezotolerans. The length of the consensus sequence (3349510 nucleotides), and the G+C content (60.21%), of LotusPond_MAG_Halomonas_sp. was comparable with the median genome lengths and median G+C contents of *H*. *johnsoniae* (3.82 mb, and 60.1%), *H*. *stevensii* (3.69 mb, and 60.2%) and *H*. *hamiltonii* (3.93 mb, and 60.1%).

**LotusPond_MAG_Unclassified_Desulfurococcales** was the only archaeal genome bin to be constrcuted from Lotus Pond’s vent-water. In default TYGS search, the MAG exhibited highest genome-genome relatedness (12.6% dDDH) with strains of the genera *Hyperthermus*, *Ignicoccus* and *Aeropyrum*, which belong to different families (Desulfurococcaceae and Pyrodictiaceae) of the order Desulfurococcales, within the archaeal class Thermoprotei. Its consensus sequence encompassed a 736-nucleotide long, partial 16S rRNA gene which showed highest sequence similarity (93.5% at 100% query coverage) with homologs from *Aeropyrum camini* as well as *Aeropyrum pernix*. The MAG also contained a complete 23S rRNA gene (3069-nucleotide long) that exhibited maximum (90.9% and 90.8%) sequence similarity at 100% query coverage with homologs from *A*. *pernix* and *A*. *camini*. The length of the consensus sequence (1553981 nucleotides), and the G+C content (49.38%), of LotusPond_MAG_Unclassified_Desulfurococcales were also comparable with the median genome lengths and median G+C contents of *Hyperthermus butylicus* (1.67 mb, and 53.7%), *Ignicoccus hospitalis* (1.3 mb, and 56.5%), *A*. *pernix* (1.64 mb, and 56.4%), *A*. *camini* (1.59 mb, and 56.7%) and other *Aeropyrum* species (1.47 mb, and 53.15%).

**LotusPond_MAG_Vibrio_metschnikovii**, in default TYGS search, exhibited highest genome-genome relatedness with strains of *Vibrio metschnikovii* and *Vibrio injensis* (85% and 77% dDDH respectively). It encompassed two 5S, but no 16S or 23S, rRNA genes. The two 5S rRNA genes annotated were 110-nucleotide and 109-nucleotide long, and possessed 99.1% mutual sequence similarity (only one substitutions across the 109 nucleotide alignment), alongside 99.1% sequence similarity (at 100% query coverage) with homologs from *Vibrio furnissii*, *Vibrio fluvialis* and/or *Vibrio tarriae*. The length of the consensus sequence (3487509 nucleotides), and the G+C content (44.28%), of LotusPond_MAG_Vibrio_metschnikovii was comparable with the median genome lengths and median G+C contents of *V*. *metschnikovii* (3.74 mb, and 44.1%), *V*. *injensis* (3.67 mb, and 44%) and other *Vibrio* species (5.09 mb, and 44.5%).

**LotusPond_MAG_Thermus_sp.**, in default TYGS search, exhibited highest genome-genome relatedness with strains of *Thermus albus* and *Thermus altitudinis* (45% and 40.1% dDDH respectively), followed by other *Thermus* species (≤40% dDDH). The 1498-nucleotide long 16S rRNA gene identified within this MAG exhibited highest (96.9% and 96.2%) sequence similarity (at 97% and 100% query coverage) with the homolog from *Thermus caldifontis* and *Thermus caldilimi* respectively. The 2876-nucleotide long 23S rRNA gene identified within this MAG also exhibited highest (96.8%) sequence similarity (at 100% query coverage) with its *Thermus caldilimi* counterpart. The 108-nucleotide long 5S rRNA gene of LotusPond_MAG_Thermus_sp. showed highest (99.1%) sequence similarity (at 100% query coverage) with the *T*. *caldilimi* homolog. The length of the consensus sequence (1887946 nucleotides), and the G+C content (62.80%), of this Lotus Pond MAG was somewhat lower than the median genome lengths and median G+C contents of *T*. *albus* (2.26 mb, and 64.3%), *T*. *altitudinis* (2.35 mb, and 65.1%), *T*. *caldifontis* (2.16 mb, and 64.8%), *T*. *thermophilus* (2.17 mb, and 69.2%), and other *Thermus* species (2.21 mb, and 66.5%). Corroborative to its genome length and G+C content delineated thus far, LotusPond_MAG_Thermus_sp. had a lower level of completeness (70.34%) and higher level of contamination (4.24%), as compared the above five Lotus Pond MAGs.

**Two *Tepidimonas*-affiliated population genome bins**, LotusPond_MAG_Tepidimonas_sp. and LotusPond_MAG_Tepidimonas_taiwanensis, were obtained from the assembled metagenome. Species level identity could not be ascertained for LotusPond_MAG_Tepidimonas_sp. having moderate to low genome-genome relatedness with different *Tepidimonas* species (in default TYGS search, highest dDDH of 45% was recorded with respect to *Tepidimonas fonticaldi* strains, while dDDH with other *Tepidimonas* species were ≤35.3%). In contrast, LotusPond_MAG_Tepidimonas_taiwanensis, in default TYGS search, showed exclusively high genome-genome relatedness (73.2% dDDH) with strains of *Tepidimonas taiwanensis*, while dDDH with other *Tepidimonas* species were ≤37%. Notably, the consensus sequences obtained thus far for the two *Tepidimonas* MAGs encompassed no 5S, 16S, or 23S rRNA gene.

Consistent with its 94.16% completeness and 1.29% contamination levels, the length of the consensus sequence (2150705 nucleotides), and the G+C content (66.30%), of LotusPond_MAG_Tepidimonas_sp. were somewhat lower than the median genome lengths and median G+C contents of its closest genomic relative *T*. *fonticaldi* (2.74 mb, and 69.5%), but comparable with those of a few other *Tepidimonas* species (2.13 mb, and 67.8%). LotusPond_MAG_Tepidimonas_taiwanensis, although potentially 90.53% complete and 3.56% contaminated, had a consensus sequence length of 2568138 nucleotides and G+C content 69.51%, which was more or less coparable with the median genome lengths and median G+C contents of its closest genomic relative *Tepidimonas* *taiwanensis* (2.85 mb, and 68.8%).

**LotusPond_MAG_Thermosynechococcus_sp.**, in default TYGS search, exhibited highest genome-genome relatedness (12.6% dDDH) with species of *Leptodesmis*, *Neosynechococcus*, *Planktothrix* and *Sodalinema*. However, the 1204-nucleotide long 16S rRNA gene identified within this MAG exhibited highest (99.6%) sequence similarity (at 100% query coverage) with the homolog from *T*. *elongatus* BP-1. The 2842-nucleotide long 23S rRNA gene identified within this MAG also exhibited highest (98.7%) sequence similarity (at 100% query coverage) with its *T*. *elongatus* BP-1 counterpart. The 110-nucleotide long 5S rRNA gene of LotusPond_MAG_Thermosynechococcus_sp. too showed highest (93.6%) sequence similarity (at 100% query coverage) with the BP-1 homolog. In all possibility, default TYGS search did not reveal any relationship of this MAG with *Thermosynechococcus* because the database used did not contain any genome sequence for members of this genus. In contrast, however, analyses using GTDB-Tk and RAST identified close relationships with the genus *Thermosynechococcus* and the strain *T*. *elongatus* BP-1 respectively. Corroborative to the above points, GGDC analysis showed that this 97.05% complete, and 0.12% contaminated, cyanobacterial MAG possesses 67.5% dDDH with the genome of *Thermosynechococcus* *elongatus* BP-1. Furthermore, the length of the consensus sequence (2328058 nucleotides), and the G+C content (52.52%), of LotusPond_MAG_Thermosynechococcus_sp. resembled only the corresponding numbers of *Thermosynechococcus* *elongatus* (2.65 mb, and 53.3%), and not those of *Leptodesmis*, *Neosynechococcus*, *Planktothrix* and *Sodalinema* species.

**LotusPond_MAG_Fervidobacterium_sp.**, in default TYGS search, exhibited highest genome-genome relatedness (42.1% to 44.0% dDDH) with strains of *Fervidobacterium pennivorans*, followed by other *Fervidobacterium* species (≤19.7% dDDH). This MAG encompassed three 5S, but no 16S or 23S, rRNA genes. Two of these had exactly identical sequences while the third copy had a single nucleotide substituted; correspondingly, each of the three 106-nucleotide long sequences individually exhibited highest (99.1% to 100%) sequence similarity (at 100% query coverage) with homologs of F. pennivorans*.* Consistent with its 70.18% completeness and 0.88% contamination levels, the length of the consensus sequence (1525481 nucleotides), and the G+C content (39.93%), of LotusPond_MAG_Fervidobacterium_sp. were somewhat different from the median genome length and median G+C content of its closest genomic relative *F*. *pennivorans* (2.06 mb, and 38.9%), but essentially comparable with other *Fervidobacterium* species (median genome length 1.67 mb, and median G+C content 39.4%).

**Two *Paracoccus*-affiliated population genome bins**, LotusPond_MAG_Paracoccus_sp._1 and LotusPond_MAG_Paracoccus_sp._2, were obtained from the assembled metagenome. Species level identity could not be ascertained for either of these two MAGs having moderate to low genome-genome relatedness with a host of *Paracoccus* species. In default TYGS search, LotusPond_MAG_Paracoccus_sp._1 exhibited hghest dDDH with strains of *Paracoccus aestuarii* (17%) and *Paracoccus hibiscisoli* (16.8%), while LotusPond_MAG_Paracoccus_sp._2 showed hghest dDDH with strains of *Paracoccus jeotgali* (15.3%) and *Paracoccus stylophorae* (15%). Furthermore, LotusPond_MAG_Paracoccus_sp._1 exhibited 14% dDDH (in GGDC-based analysis) and 73.57% orthologous gene-based ANI with the genome of the thermo-endurant *Paracoccus* strain SMMA_5, which was isolated and characterized previously from Lotus Pond’s vent-water (Roy et al., 2016; Mondal et al., 2022); LotusPond_MAG_Paracoccus_sp._2 also exhibited 15.3% GGDC-based dDDH and 75.91% orthologous gene-based ANI with SMMA_5. Notably, the consensus sequences obtained thus far for the two *Paracoccus* MAGs encompassed no 5S, 16S, or 23S rRNA gene.

The 2138329-nucleotide long LotusPond_MAG_Paracoccus_sp._1 had the thrid lowest completeness (51.22%), and the highest level of contamination (15.08%), among the 15 bins shortlisted by DASTool. In contrast, the relatively smaller (1087115-nucleotide long and 34.31% complete) LotusPond_MAG_Paracoccus_sp._2 had a contamination level of only 1.1%. Notwithstanding their low completeness levels, G+C content of both the *Paracoccus* MAGs (62.94% and 64.14% respectively) was not only within the range (61.1% to 70.7%), but also quite close to the median value (66.8%), of the G+C contents known for all sequenced *Paracoccus* genomes.

**LotusPond_MAG_Unclassified_Bacteria**, in default TYGS search, exhibited equivalently low genome-genome relatedness (12.5% dDDH) with diverse species from distinct phyla such as Proteobacteria (*Rugamonas aceris*) and Actinomycetota (*Curtobacterium herbarum*). The 2025372-nucleotide long draft genome of this potentially novel phylum level entity had a G+C content of 61.84%, while its completeness and contamination levels were 81.86% and 0.31% respectively.

**LotusPond_MAG_Unclassified_Chromatiales**, in default TYGS search, exhibited highest genome-genome relatedness (12.9% dDDH) with strains of *Thioalkalivibrio* and *Halomonas*, which belong to different orders (Chromatiales and Oceanospirillales respectively) of the class Gammaproteobacteria. This MAG, however, encompassed a 785-nucleotide long, partial 16S rRNA gene, which showed highest (98.3%) sequence similarity (at 97% query coverage) with the homolog from *Thiofaba tepidiphila* BDA453^T^, the type strain of the monotypic genus *Thiofaba*, under the family Halothiobacillaceae, within the order Chromatiales. As for its relations with the other two genera of Halothiobacillaceae, the 16S rRNA gene of LotusPond_MAG_Unclassified_Chromatiales had only ~87% sequence similarity (at 100% query coverage) with the homologs belonging to *Halothiobacillus* species, and ~88% sequence similarity (at 90% query coverage) with the 16S rRNA gene of *Thiovirga sulfuroxydans* SO07^T^ the type strain of the monotypic genus *Thiovirga*. Notably, pairwise16S rRNA gene sequence similarity between the type strains of all known Halothiobacillaceae species, namely, *Halothiobacillus neapolitanus*, *Halothiobacillus kellyi*, *T*. *tepidiphila* and *T*. *sulfuroxydans* ranged between 85.06% and 92.45% (at 95% to 98% query coverage), i.e. much lower than the similarity recorded between LotusPond_MAG_Unclassified_Chromatiales and *T*. *tepidiphila* BDA453^T^. Incidentally, *T*. *tepidiphila*, the phylogenetically closeset relative of LotusPond_MAG_Unclassified_Chromatiales, does not have a sequenced genome reported in the literature, but *H*. *neapolitanus* has one. When TYGS analysis was carried out by manually selecting *H*. *neapolitanus* DSM 15147^T^ as the target sequence from the curated database, only 12.6% dDDH was recorded in terms of “(total HSP length) / (TGL)”; however, in terms of the index “(total identities found in HSPs) / (total HSP length)”, highest (17.9%) dDDH for LotusPond_MAG_Unclassified_Chromatiales was recorded with *H*. *neapolitanus* DSM 15147^T^ as well as a number of species belonging to *Thioalkalivibrio* (family Ectothiorhodospiraceae within the order Chromatiales) but not *Halomonas*. Furthermore, the length of the consensus sequence (1644354 nucleotides), and the G+C content (62.57%), of the 91.04% complete, and 2.01% contaminated, LotusPond_MAG_Unclassified_Chromatiales resembled the corresponding numbers of neither *H*. *neapolitanus* DSM 15147^T^ (2.59 mb, and 54.70%), nor *Thioalkalivibrio sulfidiphilus* HL-EbGr7 (3.46 mb, and 65.10%) and *Thioalkalivibrio denitrificans* ALJD (3.64 mb, and 64.6%).

**LotusPond_MAG_Unclassified_Thermodesulfobacteriaceae**, in default TYGS search, exhibited highest genome-genome relatedness with species of *Thermodesulfobacterium* (12.8% dDDH) and *Caldimicrobium* (12.7% dDDH), while relatedness with species outside the family Thermodesulfobacteriaceae (or for that matter the monotypic phylum Thermodesulfobacteria) were lower. Notably, this 439694-nucleotide long MAG had the lowest completeness (30.15%) among the 15 bins reported, but its contamination level (0.31%) was quite low; its G+C content (36.23%) was also comparable with the median G+C contents of *Thermodesulfobacterium* (36.4%) and *Caldimicrobium* species (38.8%).

**Metabolically specialized (environmentally advantageous) functions encoded by the Lotus Pond MAGs**

Majority of the CDSs revealed within the Lotus Pond MAGs were attributed to basic house-keeping functions, but on top of that each population genome encoded considerable number of specialized metabolic functions that could confer the organism adaptive advantages within the hydrothermal environment.

**LotusPond_MAG_Unclassified_Aquificaceae** contained a total of 1754 putative genes, of which 1707 were protein coding sequences. 24 of these CDSs managed various cellular distress, including oxidative stress, periplasmic stress, and carbon starvation; 7 CDSs were attributed to heat stress management at the molecular level; 20 were involved in membrane transport functions such as protein translocation using the twin-arginine motif, cation transport, and those involving Ton and Tol transport systems; 15 enabled invasion and intracellular resistance; 14 conferred flagellar motility; 9 conferred resistance to toxic susbstances such as copper, cobalt, zinc and cadmium; 9 helped in CO_2_ fixation; 6 helped in lactate fermentation; 4 metabolized one-carbon compounds using tetrahydropterines; 2 governed stringent response to environmental stimuli via metabolism of guanosine tetraphosphate and pentaphosphate that are collectively called (p)ppGpp; and 1 CDS encoded a functionally versatile sporulation-associated protein. The MAG also contained three CRISPR arrays, together with 30 CRISPR repeats, and 27 CRISPR spacers.

**LotusPond_MAG_Sulfurihydrogenibium_azorense** encompassed a total of 1680 putative genes, of which 1639 were protein coding sequences. 20 of these CDSs managed various kinds of cellular distress, including oxidative stress, nitrosative stress, uptake and detoxification of selenate and selenite, periplasmic stress, and high frequency of lysogenization; 9 CDSs were attributed to heat stress management; 19 CDSs conferred flagellar motility; 15 were involved in membrane transport functions such as protein translocation using the twin-arginine motif, cation transport, and those involving Ton and Tol transport systems; 15 enabled invasion and intracellular resistance; 11 conferred resistance to toxic susbstances such as copper, cobalt, zinc, cadmium, beta-lactam antibiotics, and various other drugs (mostly via efflux pumps); 4 metabolized one-carbon compounds using tetrahydropterines; 3 helped in lactate fermentation; 2 governed stringent response to environmental stimuli via metabolism of (p)ppGpp; and 1 CDS each encoded a functionally versatile sporulation-associated protein and a toxin-antitoxin module protein. This MAG also contained 10 CRISPR arrays, together with 97 CRISPR repeats, and 87 CRISPR spacers.

**LotusPond_MAG_Halomonas_sp.** encompassed 3245 putative genes, of which 3176 were protein coding sequences. 84 CDSs managed various kinds of cellular distress, including osmotic stress, nitrosative stress, oxidative stress, uptake and detoxification of selenate / selenite and formaldehyde, periplasmic stress, carbon starvation, and high frequency of lysogenization; 15 CDSs were attributed to heat stress management; 65 conferred flagellar motility; 78 were involved in membrane transport functions such as protein translocation using the twin-arginine motif, cation transport, Ton and Tol transport systems, ABC transporters, Uni- Sym- and Antiporters, TRAP transporters, and Type IV (protein and nucleoprotein) secretion system; 12 enabled invasion and intracellular resistance; 33 genes were concerned with encoding proteins of different toxin-antitoxin modules; 30 conferred resistance to toxic susbstances such as copper, cobalt, zinc, cadmium, chromium, and various drugs (mostly via efflux pumps); 53 helped in the mixed acid fermentations, butanol biosynthesis, lactate and acetyl-CoA fermentation, acetoin and butanediol metabolism; 5 metabolized one-carbon compounds using tetrahydropterines; 3 governed stringent response to environmental stimuli via metabolism of (p)ppGpp; and 3 helped in the formation of persister cells while 1 CDS encoded a functionally versatile sporulation-associated protein. This MAG also contained 2 CRISPR arrays, together with 29 CRISPR repeats, and 27 CRISPR spacers.

**LotusPond_MAG_Unclassified_Desulfurococcales** contained 1621 putative genes, of which 1569 were protein coding sequences. 2 CDSs managed oxidative stress, 1 each managed nitrosative stress and carbon starvation; only 2 CDSs were putatively attributed to heat stress management; 1 conferred chemotaxis; 16 were involved in protein translocation using the twin-arginine motif, cation transport, ABC transporters, and TRAP transporters; 3 conferred resistance to copper, cobalt, zinc and cadmium; 13 helped in butanol biosynthesis, lactate and acetyl-CoA fermentation; and 2 governed cAMP signaling. This MAG also contained 1 CRISPR array, together with 72 CRISPR repeats, and 71 CRISPR spacers.

**LotusPond_MAG_Vibrio_metschnikovii** encompassed 3222 putative genes, of which 3172 encoded proteins. 64 CDSs managed diverse cellular distress, including osmotic stress, oxidative stress, nitrosative stress, sugar-phosphate stress, uptake and detoxification of selenate / selenite and formaldehyde, periplasmic stress, carbon starvation, and high frequency of lysogenization; 12 CDSs were attributed to heat stress management; 78 conferred flagellar motility; 74 were involved in membrane transport functions such as protein translocation using the twin-arginine motif and Type II secretion system, cation transport, Ton and Tol transport systems, ABC transporters, Uni- Sym- and Antiporters, TRAP transporters, and Type IV secretion system; 4 synthesized cholera toxin; 15 enabled invasion and intracellular resistance; 26 conferred resistance to susbstances potentially toxic to the bacterium, e.g. copper, cobalt, zinc, cadmium, chromium, mercury, colicin E2, and various antimicrobial drugs tha are resisted against by the help of efflux pumps; 16 helped in lactate fermentation, and acetoin and butanediol metabolism; 26 metabolized one-carbon compounds via serine-glyoxylate cycle or tetrahydropterines; around 60 genes were involved in the regulation of cellular response via cAMP signaling, LysR-family proteins, CytR regulation, orphan regulatory proteins, (p)ppGpp metabolism, quorum sensing and biofilm formation, autoinducer 2 (AI-2) transport and processing, TyrR-mediated virulence regulation, murein hydrolase regulation and cell death, toxin-antitoxin replicon stabilization systems, or Phd-Doc / YdcE-YdcD toxin-antitoxin (programmed cell death) systems (overall 19 genes were concerned with encoding different toxin-antitoxin modules); and 1 CDS encoded a functionally versatile sporulation-associated protein.

**LotusPond_MAG_Thermus_sp.** encompassed 2206 putative genes, of which 2157 were protein coding sequences. 6 CDSs managed osmotic, oxidative, and periplasmic stress; 7 CDSs were attributed to heat stress management; 4 rendered ammonia assimilation and reductive denitrification; 15 were involved in governing cation transport, Uni- Sym- and Antiporters, and TRAP transporters; 12 were concerned with invasion and intracellular resistance; 17 conferred resistance to toxic susbstances such as copper, cobalt, zinc and cadmium; 20 helped in butanol biosynthesis, and lactate and acetyl-CoA fermentation; 6 metabolized one-carbon compounds using tetrahydropterines; 12 genes were concerned with encoding proteins of different toxin-antitoxin modules; and 8 were involved in the regulation of cellular response via cAMP signaling or (p)ppGpp metabolism. This MAG also contained 5 CRISPR arrays, together with 43 CRISPR repeats, and 38 CRISPR spacers.

**LotusPond_MAG_Tepidimonas_sp.** encompassed 2200 putative genes, of which 2155 were protein coding sequences. 47 CDSs managed various kinds of cellular distress, including osmotic stress, oxidative stress, general cytotoxicity, periplasmic stress, and high frequency of lysogenization; 12 CDSs were attributed to heat stress management; 62 conferred flagellar motility; 6 were related to transposable elements; 9 rendered nitrate and nitrite ammonification; 62 were involved in membrane transport functions such as protein translocation using the twin-arginine motif, cation transport, Ton and Tol transport systems, Uni- Sym- and Antiporters, TRAP transporters, and Type IV secretion system; 15 enabled invasion and intracellular resistance; 24 conferred resistance to toxic susbstances such as copper, cobalt, zinc and cadmium; 31 helped in butanol biosynthesis, and lactate and acetyl-CoA fermentation; 18 governed CO_2_ fixation; 5 metabolized one-carbon compounds using tetrahydropterines; 6 were involved in the regulation and cell signaling via LysR-family proteins or (p)ppGpp metabolism; 4 genes were concerned with encoding proteins of toxin-antitoxin modules; and 1 CDS encoded a functionally versatile sporulation-associated protein. This MAG also contained 3 CRISPR arrays, together with 17 CRISPR repeats, and 14 CRISPR spacers.

**LotusPond_MAG_Fervidobacterium_sp.** encompassed 1639 putative genes, of which 1611 were protein coding sequences. 12 CDSs managed various kinds of cellular distress, including osmotic stress, oxidative stress, general cytotoxicity, periplasmic stress, and high frequency of lysogenization; 6 CDSs were attributed to heat stress management; 3 were related to cation transporters and ABC transporters; 9 enabled invasion and intracellular resistance; 7 conferred resistance to toxic susbstances such as copper, cobalt, zinc, cadmium, and various drugs (via efflux pumps); 2 helped in lactate fermentation; 5 metabolized one-carbon compounds using tetrahydropterines; 3 genes were concerned with encoding proteins of toxin-antitoxin modules; 2 governed stringent response via (p)ppGpp metabolism; and 1 CDS encoded a sporulation-associated protein. This MAG also contained 1 CRISPR array, together with 18 CRISPR repeats, and 17 CRISPR spacers.

**LotusPond_MAG_Thermosynechococcus_sp.** encompassed 2367 putative genes, of which 2319 were protein coding sequences. 34 CDSs managed various kinds of cellular distress, including oxidative stress, general cytotoxicity, and periplasmic stress; 16 CDSs were attributed to heat stress management; 22 were involved in membrane transport functions such as protein translocation using the twin-arginine motif, cation transport, Ton and Tol transport systems, and Type IV secretion system; 10 enabled invasion and intracellular resistance; 7 conferred resistance to toxic susbstances such as copper, cobalt, zinc and cadmium; 14 helped in the mixed acid fermentations, and lactate fermentation; 14 governed CO_2_ fixation; 5 metabolized one-carbon compounds using tetrahydropterines; 15 managed the Cyanobacterial Circadian Clock; 5 regulated cellular response via cAMP signaling or (p)ppGpp metabolism, while 1 enabled programmed cell death using bacterial caspases; 1 CDS each encoded a functionally versatile sporulation-associated protein and a toxin-antitoxin module protein.

**LotusPond_MAG_Tepidimonas_taiwanensis** encompassed 2567 putative genes, of which 2525 were protein coding sequences. 37 CDSs managed osmotic stress, oxidative stress, nitrosative stress uptake and detoxification of selenate and selenite, general cytotoxicity, periplasmic stress, and high frequency of lysogenization; 8 CDSs were attributed to heat stress management; 17 conferred flagellar motility; 9 encoded small phage-like gene transfer elements capable of packaging and transferring host DNA, while 2 coded for phage capsid proteins; 43 were involved in membrane transport functions such as protein translocation using the twin-arginine motif, cation transport, Ton and Tol transport systems, Uni- Sym- and Antiporters, and TRAP transporters; 6 enabled invasion and intracellular resistance; 13 conferred resistance to toxic susbstances such as copper, cobalt, zinc, cadmium and chromium; 30 helped in butanol biosynthesis, and lactate and acetyl-CoA fermentation; 19 governed CO_2_ fixation; 5 metabolized one-carbon compounds using tetrahydropterines; 6 were involved in the regulation and cell signaling via LysR-family proteins or (p)ppGpp metabolism, while 8 and 4 governed toxin-antitoxin replicon stabilization systems, and Phd-Doc / YdcE-YdcD toxin-antitoxin (programmed cell death) systems, respectively (apart from these, there were 22 more genes concerned with encoding proteins of other toxin-antitoxin modules); 1 CDS encoded a functionally versatile sporulation-associated protein. This MAG also contained 3 CRISPR arrays, together with 33 CRISPR repeats, and 30 CRISPR spacers.

**LotusPond_MAG_Paracoccus_sp._1** encompassed 2383 putative genes, of which 2345 were protein coding sequences. 55 CDSs managed various kinds of cellular distress, including osmotic stress, oxidative stress, nitrosative stress, formaldehyde toxicity, periplasmic stress, and high frequency of lysogenization; 10 CDSs were attributed to heat stress management; 3 CDSs coded for phage capsid proteins; 31 were involved in membrane transport functions such as protein translocation using the twin-arginine motif, cation transport, ABC transporters, and TRAP transporters; 3 enabled invasion and intracellular resistance; 43 conferred resistance to toxic susbstances such as arsenic, mercury, copper, cobalt, zinc, cadmium, etc.; 28 helped in butanol biosynthesis, and lactate and acetyl-CoA fermentation; 4 metabolized one-carbon compounds using tetrahydropterines; 4 CDSs were involved in the regulation and cell signaling via LysR-family proteins or (p)ppGpp metabolism, while 3 and 5 could be attributed to murein hydrolase regulation and cell death, and different toxin-antitoxin modules, respectively; and 1 CDS encoded a functionally versatile sporulation-associated protein.

**LotusPond_MAG_Unclassified_Bacteria** encompassed 2082 genes, of which 2039 encoded putative proteins. 8 CDSs managed various kinds of cellular distress, including general cytotoxicity, carbon starvation, and high frequency of lysogenization; 8 CDSs were attributed to heat stress management; 6 were involved in membrane transport functions such as cation transport, ABC transporters, and Uni- Sym- and Antiporters; 6 enabled invasion and intracellular resistance; 10 conferred resistance to toxic susbstances such as copper, cobalt, zinc, cadmium, etc., along with vancomycin and beta-lactam antibiotics; 1 helped in lactate fermentation; 3 metabolized one-carbon compounds using tetrahydropterines; 2 CDSs were involved in the regulation and cell signaling via (p)ppGpp metabolism; 5 genes were concerned with encoding proteins of toxin-antitoxin modules; and 2 CDS encoded functionally versatile sporulation-associated proteins.

**LotusPond_MAG_Unclassified_Chromatiales** encompassed 1796 putative genes, of which 1758 were protein coding sequences. 32 CDSs managed various kinds of cellular distress, including osmotic stress, oxidative stress, nitrosative stress, general cytotoxicity, periplasmic stress, and carbon starvation; 11 CDSs were attributed to heat stress management; 42 were involved in membrane transport functions such as protein translocation using the twin-arginine motif or the bacterial signal recognition particle, cation transport, Ton and Tol transport systems, Uni- Sym- and Antiporters, and TRAP transporters; 14 enabled invasion and intracellular resistance; 8 conferred resistance to toxic susbstances such as copper, zinc, etc., along with beta-lactam antibiotics; 3 helped in lactate fermentation; 2 metabolized one-carbon compounds using tetrahydropterines; 11 governed CO_2_ fixation; 4 CDSs were involved in the regulation and cell signaling via LysR-family proteins or (p)ppGpp metabolism, while 9 could be attributed to different toxin-antitoxin systems; and 1 CDS encoded a functionally versatile sporulation-associated protein.

**LotusPond_MAG_Paracoccus_sp._2**, evidently due to its low level of completeness (34.3%), encompassed only 1214 putative genes, of which 1201 were protein coding sequences. 22 CDSs managed oxidative stress, formaldehyde toxicity, and periplasmic stress; 8 enabled reductive denitrification; 6 CDSs were attributed to heat stress management; 1 CDS was linked to a phage packaging machinery; 20 governed cation transport, Ton and Tol transport systems, and TRAP transporters; 4 enabled invasion and intracellular resistance; 6 conferred resistance to copper, colicin E2, etc.; 9 helped in lactate and acetyl-CoA fermentation; 4 metabolized one-carbon compounds using tetrahydropterines; 2 CDSs were involved in regulation and cell signaling via (p)ppGpp metabolism, while 2 governed a toxin-antitoxin system.

**LotusPond_MAG_Unclassified_Thermodesulfobacteriaceae**, also because of very low completeness level (30.2%), encompassed only 477 putative genes, of which 467 were protein coding sequences. 2 CDSs managed periplasmic stress; only 3 CDSs were putatively attributed to heat stress management; 7 enabled invasion and intracellular resistance; 6 metabolized one-carbon compounds using tetrahydropterines; and 2 CDSs were involved in the regulation and cell signaling by (p)ppGpp metabolism.

**Metagenome-encoded functions that can help bacterial mesophiles survive in the Lotus Pond habitat**

Lotus Pond’s vent-water metagenome encompassed a large repertoire of genes encoding proteins that have the potentials for protecting microorganisms from physicochemically challenging environmental conditions, including high *in situ* heat. Many of these genes seemed to have decisive roles in thermal adaptation in the context of Lotus Pond’s geochemistry and topography. Furthermore, a majority of these CDSs were ascribable to taxa having no cultured strain reported for *in vitro* growth at temperatures near the Lotus Pond vent-water temperature (Table S2). A few examples of such ecologically advantageous genes having large numbers of homologs in the assembled metagenome are given below.

1. >550 and >1550 genes encoding heat shock proteins and molecular chaperones that render the quality control of DNA and proteins amid biophysical stress respectively. Some of the most copious of these genes were the ones encoding the 70 kDa heat shock protein DnaK (~250 homologs detected) and subunits of caseinolytic protease (Clp) that rescues other proteins from aggregated states (~500 homologs).
2. ~150 genes encoding various subunits of the GroEL/GroES chaperonin complex.
3. >150 genes encoding proteins that control/modulate/inhibit the activities of DNA gyrase (or its inhibitor molecules), as opposed to <100 genes for DNA gyrase / Type IIA topoisomerase / Topoisomerase IV itself. In microorganisms growing at boiling or near-boiling temperatures, i.e. in true thermophiles, active reverse gyrases (RGs) are indispensible as they render positive supercoils in the DNA and counter-balance the promotion of negative supercoiling by not only gyrase enzymes but also the high *in situ* heat (López-García, 1999; Lipscomb et al., 2017). Furthermore, RGs help avert incorrect aggregation of denatured DNA segments and facilitate correct annealing via actions analogous to protein chaperones (Kampmann and Stock, 2004). Expectedly, the Lotus Pond MAGs affiliated to Aquificaceae, Desulfurococcales, *Fervidobacterium* and *Sulfurihydrogenibium* encompassed RGs, while the assembled metagenome also contained gyrase modulators ascribable to Aquificae and Crenarchaeota. On the other hand, the MAGs belonging to phylogenetic relatives of mesophilic bacteria were devoid of RGs, but those affiliated to *Halomonas*, *Vibrio*, *Tepidimonas* and *Paracoccus* did encompass genes encoding gyrase inhibitors/modulators such as YacG/PmbA/TldD which are known to nullify DNA gyrase activity in bacterial mesophiles (Murayama et al., 1996; Sengupta and Nagaraja, 2008); several homologs of these genes ascribable to diverse mesophilic taxa were also present in the assembled metagenome. Future studies of pure-culture transcriptomics and proteomics are needed to reveal the potential controls of expressions, activities and interactions of gyrases and anti-gyrases that help hot spring mesophiles modulate DNA metabolism in response to thermal stress.
4. At least 276 genes encoding different toxin–antitoxin (TA) systems, which act as regulatory modules for growth and survival of bacterial cells under diverse stress conditions (Page and Peti, 2016). Most of the genes encoding TA systems were concerned with the synthesis of the various modules of the bacterial (ribonuclease-based) Type II system. Considerable numbers of genes were also there for endonuclease- / endoribonuclease-based TA systems, Type IV TA systems, the RelE / RelB, PrlF / YhaV, YdaS / YdaT, HicAB, and MraZ / MraW systems.
5. ~175 genes encoding universal stress proteins, homologs of which are also possessed by bacterial mesophiles occupying ecological niches adjacent to deep sea hydrothermal vent (Hasan et al., 2015).
6. ~100 encoding methionine sulfoxide reductases (Msr), which repair oxidatively damaged or inactivated proteins by converting their methionine sulfoxides to methionines.
7. ~450 genes encoding enzymes known to protect cells from the toxic effects of peroxides, which in turn are often abundant in hot spring waters (Meslé et al., 2017). Examples included CDSs for alkyl hydroperoxide reductase and alkyl hydroperoxidase, glutathione peroxidase, bifunctional enzymes having both catalase and broad- spectrum peroxidase activities, cytochrome c peroxidase, di-haem cytochrome c peroxidase, Dyp-type peroxidase, thiol-specific peroxidase, superoxide dismutase, iron/manganese superoxide dismutase, and organic hydroperoxide resistance proteins.
8. ~850 genes for diverse proteins adapted structurally and/or functionally to high concentrations of heavy metals, which is a common feature of hydrothermal discharges (Mulkidjanian et al., 2012; Sherpa et al., 2013; Van Kranendonk et al., 2021; Jawadi et al., 2021) including those of Lotus Pond and other hot springs of Puga Valley (Garrett, 1998; Rai et al., 2001; Ghosh et al., 2012; Roy et al., 2020a, 2020b). Examples comprised CDSs for various kinds of metallopeptidases; metalloproteases including aminopeptidases, zinc metalloproteases, elastases and thermophilic metalloproteases of the M29 family; metallochaperones; metallophosphoesterases; metallo-endoribonucleases; chaperone/metalloprotease dual-function proteins that promote the assembly or elimination of other proteins depending on the latters’ correct or wrong folding state; proteins rendering the incorporation of metallocenters in other proteins; and diverse members of the metallo-beta-lactamase superfamily. The most copious of these genes were the ones encoding the energy-dependent zinc-metallopeptidase/protease FtsH (~120 homologs detected), essential for the quality control of both cytoplasmic and membrane proteins, alongside regulation of lipopolysaccharide biosynthesis and heat shock response.
9. ~40 genes encodING different Mnt proteins that render cellular transport and homeostasis of manganese, a key cofactor of different enzymes protecting against oxidative damage.
10. ~100 fatty acid desaturase genes which could be crucial for increasing the rigidity of cell membranes (via unsaturation of fatty acids) in the face of the high entropic effects of heat (Vezzi, et al., 2005; Hasan et al., 2015).
11. >1550 genes for flagellar structures and functions, ~480 genes for chemotaxis, >100 genes for cell adhesion and/or biofilm formation, ~40 genes for Lux-proteins-mediated quorum-sensing-based response to environmental conditions. These genes could be crucial for the *in situ* microorganisms to dynamically position themselves at their physicochemically best-suited niches along the hydrothermal gradients occurring in the vent (Lotus Pond) to river (Rulang) trajectory (Ghosh et al., 2012; Roy et al., 2020a).

**Supplementary Tables**

**Table S1. Summary of the metagenomic sequence dataset generated for Lotus Pond’s vent-water using Novaseq 6000 (Illumina Inc.) next-generation DNA sequencer.**

| **Sample name** | **No. of read-pairs** | **No. of reads** | **Total no. of bases** | **Median of quality value** | **Bases quality value ≥20** | **GC content** | **Median of read length** |
| --- | --- | --- | --- | --- | --- | --- | --- |
| Raw sequence | 31353866 | 62707732 | 15676933000 | 37 | 14254492909 | 47.5 | 250 |
| Post quality filtering | 31342225 | 62684450 | 14254492909 | 37 | 14254492909 | 45 | 249 |

**Table S3.** Taxonomic affiliation (up to the order level) of the archaeal and bacterial protein-coding sequences (CDSs) predicted within the assembled metagenome of Lotus Pond’s vent-water.

| **Phylum (no. of CDSs ascribed to the phylum)** | **Class (no. of CDSs ascribed to the class)** | **Order (no. of CDSs ascribed to the order)** |
| --- | --- | --- |
| **Archaea (total CDSs: 2309)** | | |
| Crenarchaeota / Thermoproteota (1479) | - | - |
| Euryarchaeota (647) | Halobacteria (177) | - |
|  | Thermococci (169) | - |
|  | Archaeoglobi (83) | - |
|  | Methanococci (60) | - |
|  | Methanomicrobia (30) | - |
|  | Methanobacteria (19) | - |
|  | Thermoplasmata (9) | - |
|  | Unclassified Euryarchaeota (100) | - |
| Unclassified Archaea (183) | - | - |
| **Bacteria (total CDSs: 214652)** | | |
| Proteobacteria / Pseudomonadota (90793) | Alphaproteobacteria (41306) | Rhodospirillales (2092) |
|  |  | Sphingomonadales (1099) |
|  |  | Caulobacterales (950) |
|  |  | Rickettsiales (235) |
|  |  | Unclassified Alphaproteobacteria (36930) |
|  | Betaproteobacteria (14394) | Rhodocyclales (3582) |
|  |  | Neisseriales (755) |
|  |  | Nitrosomonadales (395) |
|  |  | Unclassified Betaproteobacteria (9662) |
|  | Gammaproteobacteria (32111) | Xanthomonadales / Lysobacterales (10039) |
|  |  | Vibrionales (6025) |
|  |  | Oceanospirillales (3940) |
|  |  | Chromatiales (1529) |
|  |  | Legionellales (253) |
|  |  | Thiotrichales (246) |
|  |  | Pasteurellales (194) |
|  |  | Methylococcales (108) |
|  |  | Aeromonadales (67) |
|  |  | Unclassified Gammaproteobacteria (9710) |
|  | Deltaproteobacteria (1145) | - |
|  | Epsilonproteobacteria (466) | - |
|  | Acidithiobacillia (106) | Acidithiobacillales (106) |
|  | Hydrogenophilia (118) | Hydrogenophilales (118) |
|  | Oligoflexia (101) | Bdellovibrionales (101) |
|  | Unclassified Proteobacteria (1046) | - |
| Aquificae / Aquificota (54151) | - | - |
| Firmicutes / Bacillota (18872) | Bacilli (10238) | - |
|  | Clostridia (7710) | - |
|  | Negativicutes (649) | - |
|  | Erysipelotrichia (73) | - |
|  | Unclassified Firmicutes (202) | - |
| Deinococcus-Thermus / Deinococcota (10591) | - | - |
| Cyanobacteria (5827) | - | - |
| Chloroflexi / Chloroflexota (4254) | Chloroflexia (3762) | - |
|  | Dehalococcoidia (10) | - |
|  | Unclassified Chloroflexi (482) | - |
| Bacteroidetes / Bacteroidota (2753) | - | - |
| Thermotogae / Thermotogota (2671) | - | - |
| Thermodesulfobacteria / Thermodesulfobacteriota (2547) | - | - |
| Actinobacteria / Actinomycetota (2357) | Rubrobacteria (46) | - |
|  | Coriobacteriia (19) | - |
|  | Acidimicrobiia (6) | - |
|  | Unclassified Actinobacteria (2286) | - |
| Planctomycetes / Planctomycetota (355) | - | - |
| Synergistetes / Synergistota (200) | - | - |
| Verrucomicrobia / Verrucomicrobiota (160) | Verrucomicrobia (78) | - |
|  | Opitutae (50) | - |
|  | Verrucomicrobiae (32) | - |
| Acidobacteria / Acidobacteriota (134) | Acidobacteriia (94) |  |
|  | Unclassified Acidobacteria (40) |  |
| Fusobacteria / Fusobacteriota (133) | - | - |
| Nitrospirae / Nitrospirota (126) | - | - |
| Chlorobi / Chlorobiota (93) | - | - |
| Spirochaetes / Spirochaetota (77) | - | - |
| Thermomicrobia / Thermomicrobiota (63) | - | - |
| Deferribacteres / Deferribacterota (43) | - | - |
| Tenericutes / Mycoplasmatota (25) | - | - |
| Gemmatimonadetes / Gemmatimonadota (21) | - | - |
| Chlamydiae / Chlamydiota (20) | - | - |
| Unclassified Bacteria (18386) | - | - |

**Table S4.** Taxonomic classification of the metagenomic reads which corresponded to archaeal and bacterial 16S rRNA gene sequences (taxa that were also identified in the present amplified 16S rRNA gene sequence based analysis are written in bold fonts).

| **Phylum (no. of 16S reads ascribed to the phylum)** | **Class** | **Genus (no. of 16S reads ascribed to the genus)** | **Upper limit of temperature (°C) for laboratory growth** | **Reference** |
| --- | --- | --- | --- | --- |
| **Archaea (total reads: 396)** | | | | |
| Crenarchaeota / Thermoproteota (396) | - | *Aeropyrum* (6) | 100 | Sako et al., 1996 |
|  | - | *Pyrobaculum* (1) | 102 | Huber et al., 1987 |
|  | - | *Stetteria* (2) | 102 | Jochimsen et al., 1997 |
| **Bacteria (total reads: 81223)** | | | | |
| Actinobacteria / Actinomycetota (21) | - | ***Brevibacterium*** (2) | 42 | Kim et al., 2013 |
|  | - | ***Corynebacterium*** (1) | 42 | Bernard et al., 2016 |
|  | - | *Ornithinimicrobium* (1) | 50 | Groth et al., 2001 |
| Aquificae / Aquificota (65853) | - | ***Hydrogenobacter*** (44547) | 85 | Takai et al., 2001 |
|  | - | *Hydrogenothermus* (2) | 80 | Stohr et al., 2001 |
|  | - | *Persephonella* (6) | 80 | Götz et al., 2002 |
|  | - | ***Sulfurihydrogenibium*** (16371) | 80 | O'Neill et al., 2008 |
|  | - | *Thermocrinis* (180) | 89 | Huber et al., 1998 |
|  | - | *Venenivibrio* (48) | 75 | Hetzer et al., 2008 |
| Armatimonadetes / Armatimonadota (2) | - | Armatimonadetes_gp5 (2) | - | - |
| Bacteroidetes / Bacteroidota (56) | - | ***Chryseobacterium*** (8) | 37 | Yang et al., 2015 |
|  | - | ***Elizabethkingia*** (2) | 37 | Kämpfer et al., 2011 |
|  | - | ***Myroides*** (1) | 37 | Vancanneyt et al., 1996 |
|  | - | ***Pedobacter*** (1) | 35 | Zhang et al., 2015 |
|  | - | ***Porphyromonas*** (2) | 37 | Shah and Collins, 1998 |
|  | - | ***Sphingobacterium*** (6) | 40 | Yabuuchi et al., 1983 |
| Chloroflexi / Chloroflexota (56) | - | ***Chloroflexus*** (54) | 59 | Gaisin et al., 2017 |
| Cyanobacteria (131) | - | **GpIIa** (1) | - | - |
|  | - | GpIV (2) | - | - |
|  | - | *Pantanalinema* (1) | - | - |
|  | - | *Prochlorothrix* (4) | 30 | Burger-Wiersma et al., 1989 |
| Deinococcus-Thermus / Deinococcota (1517) | - | ***Thermus*** (1454) | 80 | Chung et al., 2000 |
| Firmicutes / Bacillota (567) | - | ***Acetoanaerobium*** (23) | 40 | Bes et al., 2015 |
|  | - | ***Aerococcus*** (2) | 37 | Williams et al., 1953 |
|  | - | ***Alkalibacterium*** (5) | 32 | Ntougias and Russell, 2001 |
|  | - | *Alkalihalobacillus* (8) | 45 | Patel and Gupta, 2020 |
|  | - | ***Anoxybacillus*** (194) | 70 | Cihan et al., 2014 |
|  | - | ***Bacillus*** (10) | 70 | Yang et al., 2013a |
|  | - | ***Enterococcus*** (48) | 45 | Schleifer et al., 1984 |
|  | - | *Granulicatella* (5) | 37 | Collins and Lawson, 2000 |
|  | - | ***Staphylococcus*** (3) | 40 | Fuente et al., 1985 |
|  | - | ***Streptococcus*** (60) | 45 | Sherman and Stark, 1931 |
|  | - | *Thermotalea* (2) | 55 | Ogg and Patel, 2009 |
| Proteobacteria / Pseudomonadota (11639) | Alphaproteobacteria | *Agrobacterium* (14) | 42 | Castellano-Hinojosa et al., 2021 |
|  |  | *Brucella* (92) | 40 | Foster et al., 2007 |
|  |  | ***Caulobacter*** (4) | 32 | Jin et al., 2013 |
|  |  | *Devosia* (2) | 45 | Ryu et al., 2008 |
|  |  | *Elioraea* (6) | 50 | Albuquerque et al., 2008 |
|  |  | ***Ensifer*** (1) | 37 | Casida Jr, 1982 |
|  |  | *Nitratireductor* (2) | 45 | Yu et al., 2016 |
|  |  | *Novosphingobium* (2) | 45 | Lee et al., 2014 |
|  |  | *Pannonibacter* (3) | 55 | Xi et al., 2018 |
|  |  | ***Paracoccus*** (196) | 45 | Sun et al., 2015 |
|  |  | *Phenylobacterium* (4) | 50 | Khan et al., 2018 |
|  |  | *Pseudochrobactrum* (2) | 40 | Romanenko et al., 2008 |
|  |  | *Rhizobium* (2) | 45 | Xu et al., 2011 |
|  |  | *Rhizorhapis* (1) | 32 | Francis et al., 2014 |
|  |  | *Sphingomonas* (2) | 45 | Gao et al., 2016 |
|  |  | *Stappia* (3) | 45 | Kämpfer et al., 2013 |
|  |  | ***Sulfitobacter*** (1) | 40 | Kumari et al., 2016 |
|  |  | *Thioclava* (1) | 47 | Sorokin et al., 2005 |
|  | Betaproteobacteria | *Acidovorax* (2) | 37 | Heylen et al., 2008 |
|  |  | *Azoarcus* (10) | 45 | Chen et al., 2013a |
|  |  | ***Burkholderia*** (1) | 37 | Aizawa et al., 2011 |
|  |  | ***Comamonas*** (1) | 44 | Chang et al., 2002 |
|  |  | *Ottowia* (2) | 37 | Felföldi et al., 2011 |
|  |  | ***Pelomonas*** (2) | 37 | Gomila et al., 2007 |
|  |  | ***Ralstonia*** (7) | 41 | Yabuuchi et al., 1995 |
|  |  | *Tepidicella* (6) | 60 | You et al., 2019 |
|  |  | ***Tepidimonas*** (965) | 60 | Chen et al., 2013b |
|  |  | ***Thauera*** (2) | 55 | Yang et al., 2018 |
|  |  | ***Uruburuella*** (15) | 37 | Vela et al., 2005 |
|  | Gammaproteobacteria | ***Aeromonas*** (4) | 41 | Huys et al., 2002 |
|  |  | *Aliivibrio* (36) | 30 | Yoshizawa et al., 2010 |
|  |  | ***Alishewanella*** (29) | 42 | Vogel et al., 2000 |
|  |  | *Arsukibacterium* (2) | 40 | Wang et al., 2022 |
|  |  | *Azomonas* (3) | - | - |
|  |  | *Buttiauxella* (2) | 42 | Müller et al., 1996 |
|  |  | *Catenococcus* (31) | 35 | Sorokin, 1992 |
|  |  | *Diplorickettsia* (5) | 28 | Mediannikov et al., 2010 |
|  |  | ***Enhydrobacter*** (2) | 41 | Staley et al., 1987 |
|  |  | ***Escherichia*/*Shigella*** (6) | 37 | Liu et al., 2015 |
|  |  | *Ferrimonas* (2) | 42 | Ji et al., 2013 |
|  |  | ***Halomonas*** (3075) | 50 | Guan et al., 2010 |
|  |  | ***Idiomarina*** (6) | 55 | Lee et al., 2015 |
|  |  | *Klebsiella* (1) | 37 | Holt, 1984 |
|  |  | ***Marinobacter*** (10) | 50 | Wang et al., 2009 |
|  |  | ***Pseudoalteromonas*** (4) | 44 | Lau et al., 2005 |
|  |  | ***Pseudomonas*** (24) | 42 | Yang et al., 2013b |
|  |  | ***Rheinheimera*** (2) | 35 | Ryu et al., 2008 |
|  |  | ***Salinivibrio*** (8) | 45 | Mellado et al., 1996 |
|  |  | ***Shewanella*** (6) | 30 | MacDonell et al., 1985 |
|  |  | ***Silanimonas*** (20) | 53 | Lee et al., 2005 |
|  |  | ***Stenotrophomonas*** (149) | 42 | Lee et al., 2011 |
|  |  | ***Thiofaba*** (75) | 51 | Mori and Suzuki, 2008 |
|  |  | ***Vibrio*** (2549) | 50 | Meng et al., 2018 |
|  |  | *Xanthomonas* (9) | 35 | Morales Nicolàs et al., 2017 |
|  |  | *Xylella* (2) | 30 | Su et al., 2016 |
| Synergistetes / Synergistota (2) | - | ***Thermanaerovibrio*** (2) | 70 | Zavarzina et al., 2000 |
| Thermodesulfobacteria / Thermodesulfobacteriota (308) | - | *Caldimicrobium* (60) | 82 | Miroshnichenko et al., 2009 |
|  | - | *Geothermobacterium* (5) | 100 | Kashefi et al., 2002 |
| Thermotogae / Thermotogota (196) | - | ***Fervidobacterium*** (193) | 90 | Cai et al., 2007 |
| Unclassified Bacteria (875) | - | - | - | - |

**Table S5.** Taxonomic classification of the OTUs obtained via PCR-amplified 16S rRNA gene sequence analysis (taxa that were also identified in the current analysis of raw metagenomic reads corresponding to 16S rRNA genes are written in bold fonts; taxa that were also identified in previous amplified 16S rRNA gene sequence based analysis have their names underlined).

| **Phylum (no. of OTUs ascribed to the phylum)** | **Class** | **Genus (no. of OTUs ascribed to the genus)** | **Upper limit of temperature (°C) for laboratory growth** | **Reference** |
| --- | --- | --- | --- | --- |
| **Archaea (total OTU count: 30)** | | | | |
| Crenarchaeota / Thermoproteota (10) | - | - | - | - |
| Euryarchaeota (6) | - | *Methanomassiliicoccus* (1) | 45 | Dridi et al., 2012 |
|  | - | *Methanospirillum* (3) | 50 | Iino et al., 2010 |
| Nitrososphaerota (6) | - | *Nitrososphaera* (6) | 47 | Stieglmeier et al., 2014 |
| Unclassified Archaea (8) | - | - | - | - |
| **Bacteria (total OTU count: 602)** | | | | |
| Acidobacteria / Acidobacteriota (2) | - | - | - | - |
| Actinobacteria / Actinomycetota (40) | - | *Boudabousia* (1) | 35 | Yang et al., 2021 |
|  | - | ***Brevibacterium*** (2) | 42 | Kim et al., 2013 |
|  | - | ***Corynebacterium*** (8) | 42 | Bernard et al., 2016 |
|  | - | *Cutibacterium* (1) | 37 | Dekio et al., 2020 |
|  | - | *Geodermatophilus* (1) | 45 | Nie et al., 2012 |
|  | - | *Mycobacterium* (1) | 43 | Kusunoki and Ezaki, 1992 |
|  | - | *Nocardioides* (1) | 37 | Prauser, 1976 |
|  | - | *Prauserella* (1) | 45 | Kim et al., 1999 |
|  | - | *Rothia* (1) | 38 | Fan et al., 2002 |
| Aquificae / Aquificota (3) | - | ***Hydrogenobacter*** (1) | 85 | Takai et al., 2001 |
|  | - | ***Sulfurihydrogenibium*** (2) | 80 | O'Neill et al., 2008 |
| Armatimonadetes / Armatimonadota (2) | - | Armatimonadetes_gp7 (2) | - | - |
| Bacteroidetes / Bacteroidota (52) | - | *Bacteroides* (2) | 45 | Sun et al., 2022 |
|  | - | ***Chryseobacterium*** (6) | 37 | Yang et al., 2015 |
|  | - | *Cloacibacterium* (1) | 40 | Chun et al., 2017 |
|  | - | ***Elizabethkingia*** (2) | 37 | Kämpfer et al., 2011 |
|  | - | *Mesonia* (1) | 40 | Zhou et al., 2021 |
|  | - | ***Myroides*** (3) | 37 | Vancanneyt et al., 1996 |
|  | - | *Nubsella* (1) | 40 | Asker et al., 2008 |
|  | - | ***Pedobacter*** (3) | 35 | Zhang et al., 2015 |
|  | - | *Pontibacter* (1) | 45 | Subhash etal., 2014 |
|  | - | ***Porphyromonas*** (2) | 37 | Shah and Collins, 1988 |
|  | - | *Prevotella* (1) | 45 | Shah and Collins, 1990 |
|  | - | *Raineya* (1) | 60 | Albuquerque et al., 2018 |
|  | - | *Schleiferia* (2) | 50 | Albuquerque et al., 2011 |
|  | - | ***Sphingobacterium*** (4) | 40 | Yabuuchi et al., 1983 |
|  | - | *Taibaiella* (1) | 37 | Kim et al., 2016 |
| Chloroflexi / Chloroflexota (2) | - | ***Chloroflexus*** (2) | 59 | Gaisin et al., 2017 |
| Cyanobacteria (20) | - | **GpIIa** (1) | - | - |
|  | - | GpXIII (1) | - | - |
| Deinococcus-Thermus / Deinococcota (16) | - | ***Thermus*** (13) | 80 | Chung et al., 2000 |
|  | - | *Truepera* (1) | 50 | Albuquerque et al., 2005 |
| Dictyoglomi / Dictyoglomota (1) | - | *Dictyoglomus* (1) | 80 | Saiki et al., 1985 |
| Firmicutes / Bacillota (63) | - | ***Acetoanaerobium*** (1) | 40 | Bes et al., 2015 |
|  | - | ***Aerococcus*** (1) | 37 | Williams et al., 1953 |
|  | - | ***Alkalibacterium*** (3) | 32 | Ntougias and Russell, 2001 |
|  | - | *Alkalicoccus* (1) | 45 | Gupta et al., 2020 |
|  | - | *Alkaliphilus* (1) | 55 | Ben Aissa et al., 2015 |
|  | - | *Amphibacillus* (1) | 55 | Ren et al., 2013 |
|  | - | *Anaerobranca* (1) | 66 | Engle et al., 1995 |
|  | - | ***Anoxybacillus*** (2) | 70 | Cihan et al., 2014 |
|  | - | ***Bacillus*** (1) | 70 | Yang et al., 2013a |
|  | - | *Chryseomicrobium* (1) | 45 | Arora et al., 2011 |
|  | - | *Cytobacillus* (1) | 37 | Zhang et al., 2010 |
|  | - | ***Enterococcus*** (4) | 45 | Schleifer et al., 1984 |
|  | - | *Exiguobacterium* (1) | 49 | Crapart et al., 2007 |
|  | - | *Fastidiosipila* (1) | 37 | Falsen et al., 2005 |
|  | - | *Gemella* (1) | 37 | Kilpper-Bälz and Schleifer, 1988 |
|  | - | *Gracilibacter* (1) | 54 | Lee et al., 2006 |
|  | - | *Halolactibacillus* (1) | 45 | Ishikawa et al., 2005 |
|  | - | *Jeotgalicoccus* (2) | 45 | Chen et al., 2009 |
|  | - | *Lacrimispora* (1) | - | - |
|  | - | *Lactococcus* (1) | 40 | Schleifer et al., 1985 |
|  | - | *Lysinibacillus* (1) | 45 | Ahmed et al., 2007 |
|  | - | *Piscibacillus* (1) | 55 | Amoozegar et al., 2009 |
|  | - | *Planococcus* (1) | 42 | Suresh et al., 2007 |
|  | - | *Romboutsia* (1) | 40 | Wang et al., 2015 |
|  | - | Sporolactobacillaceae_incertae_sedis (1) | - | - |
|  | - | ***Staphylococcus*** (3) | 40 | Fuente et al., 1985 |
|  | - | ***Streptococcus*** (3) | 45 | Sherman and Stark, 1931 |
|  | - | *Terribacillus* (1) | 45 | An et al., 2007 |
| Proteobacteria / Pseudomonadota (352) | Alphaproteobacteria | *Azospirillum* (1) | 37 | Mehnaz et al., 2007 |
|  |  | *Afifella* (1) | 35 | Buddhi et al., 2020 |
|  |  | *Bradyrhizobium* (1) | 37 | Araújo et al., 2017 |
|  |  | *Brevundimonas* (1) | 42 | Choi et al., 2010 |
|  |  | ***Caulobacter*** (1) | 32 | Jin et al., 2013 |
|  |  | ***Ensifer*** (1) | 37 | Casida Jr, 1982 |
|  |  | *Methylocystis* (1) | 40 | Bowman et al., 1993 |
|  |  | *Methylorubrum* (1) | 43 | Green and Ardley, 2018 |
|  |  | ***Paracoccus*** (1) | 45 | Sun et al., 2015 |
|  |  | *Rhodobaca* (1) | 35 | Boldareva et al., 2008 |
|  |  | *Roseibium* (1) | 45 | Duan et al., 2020 |
|  |  | *Sphingobium* (1) | 37 | Kumari et al., 2009 |
|  |  | ***Sulfitobacter*** (1) | 40 | Kumari et al., 2016 |
|  | Betaproteobacteria | *Achromobacter* (1) | 42 | Vandamme et al., 2016 |
|  |  | *Alcaligenes* (1) | 45 | Lu et al., 2017 |
|  |  | ***Burkholderia*** (1) | 37 | Aizawa et al., 2011 |
|  |  | *Chitinibacter* (1) | 40 | Chern et al., 2004 |
|  |  | ***Comamonas*** (2) | 44 | Chang et al., 2002 |
|  |  | *Delftia* (1) | 40 | Chen et al., 2012 |
|  |  | *Herbaspirillum* (1) | 45 | Fan et al., 2018 |
|  |  | *Kerstersia* (1) | 42 | Vandamme et al., 2012 |
|  |  | ***Pelomonas*** (1) | 37 | Gomila et al., 2007 |
|  |  | ***Ralstonia*** (2) | 41 | Yabuuchi et al., 1995 |
|  |  | ***Tepidimonas*** (6) | 60 | Chen et al., 2013b |
|  |  | ***Thauera*** (1) | 55 | Yang et al., 2018 |
|  |  | ***Uruburuella*** (2) | 37 | Vela et al., 2005 |
|  | Gammaproteobacteria | ***Aeromonas*** (3) | 41 | Huys et al., 2002 |
|  |  | *Acinetobacter* (11) | 37 | Smet et al., 2014 |
|  |  | *Aliidiomarina* (2) | 45 | Chiu et al., 2014 |
|  |  | ***Alishewanella*** (4) | 42 | Vogel et al., 2000 |
|  |  | ***Enhydrobacter*** (1) | 41 | Staley et al., 1987 |
|  |  | ***Escherichia*/*Shigella*** (1) | 37 | Liu et al., 2015 |
|  |  | *Guyparkeria* (1) | 49 | Boden, 2017 |
|  |  | ***Halomonas*** (16) | 50 | Guan et al., 2010 |
|  |  | ***Idiomarina*** (2) | 55 | Lee et al., 2015 |
|  |  | *Legionella* (2) | 45 | Palmer et al., 2016 |
|  |  | ***Marinobacter*** (2) | 50 | Wang et al., 2009 |
|  |  | *Morganella* (1) | 35 | Emborg et al., 2006 |
|  |  | *Nitrincola* (1) | 40 | Zhang et al., 2020 |
|  |  | ***Pseudoalteromonas*** (1) | 44 | Lau et al., 2005 |
|  |  | ***Pseudomonas*** (5) | 42 | Yang et al., 2013b |
|  |  | *Pseudoxanthomonas* (1) | 50 | Rani et al., 2010 |
|  |  | *Psychrobacter* (2) | 38 | Maruyama et al., 2000 |
|  |  | ***Rheinheimera*** (1) | 35 | Ryu et al., 2008 |
|  |  | *Rhodanobacter* (1) | 40 | Koh et al., 2015 |
|  |  | ***Salinivibrio*** (1) | 45 | Mellado et al., 1996 |
|  |  | *Serratia* (1) | 35 | Geiger et al., 2010 |
|  |  | ***Shewanella*** (1) | 30 | MacDonell et al., 1985 |
|  |  | ***Silanimonas*** (1) | 53 | Lee et al., 2005 |
|  |  | ***Stenotrophomonas*** (4) | 42 | Lee et al., 2011 |
|  |  | ***Thiofaba*** (3) | 51 | Mori and Suzuki, 2008 |
|  |  | *Thiovirga* (1) | 34 | Ito et al., 2005 |
|  |  | ***Vibrio*** (21) | 50 | Meng et al., 2018 |
|  | Deltaproteobacteria | *Desulfohalovibrio* (1) | 47 | Spring et al., 2019 |
|  | Epsilonproteobacteria | *Aliarcobacter* (1) | 42 | Pérez-Cataluña et al., 2019 |
|  | Oligoflexia | *Bdellovibrio* (1) | 30 | Koval et al., 2013 |
| Rhodothermaeota / Rhodothermota (1) | - | - | - | - |
| Spirochaetes / Spirochaetota (2) | - | *Leptospira* (1) | 30 | Faine and Stallmn, 1982 |
| Synergistetes / Synergistota (1) | - | ***Thermanaerovibrio*** (1) | 70 | Zavarzina et al., 2000 |
| Thermodesulfobacteria / Thermodesulfobacteriota (2) | - | - | - | - |
| Thermotogae / Thermotogota (8) | - | ***Fervidobacterium*** (7) | 90 | Cai et al., 2007 |
|  | - | *Oceanotoga* (1) | 70 | Jayasinghearachchi et al., 2011 |
| Unclassified Bacteria (35) | - | - | - | - |

**Supplementary References**

**References used in Supplementary Method and Supplementary Results**

Absher M. Hemocytometer counting. In Kruse PF, Patterson MK (eds). Tissue culture. Academic Press, 1973. pp 395-397.

Dong L, Qi J, Shao C, Zhong X, Gao D, Cao W, et al. Concentration and size distribution of total airborne microbes in hazy and foggy weather. Sci Total Environ. 2016;541:1011-1018.

Garrett DE. Borates: Handbook of deposits, processing, properties, and use. Academic Press, 1998.

Ghosh W, Mallick S, Haldar PK, Pal B, Maikap SC, Gupta SK. Molecular and cellular fossils of a mat-like microbial community in geothermal boratic sinters. Geomicrobiol J. 2012;29:879-885.

Hasan NA, Grim CJ, Lipp EK, Rivera IN, Chun J, Haley BJ, et al. Deep-sea hydrothermal vent bacteria related to human pathogenic *Vibrio* species. Proc Natl Acad Sci USA. 2015;112:E2813-E2819.

Jawadi HA, Malistani HA, Moheghy MA, Sagin J. Essential trace elements and arsenic in thermal springs, Afghanistan. Water. 2021;13:134.

Jones KH, Senft JA. An improved method to determine cell viability by simultaneous staining with fluorescein diacetate-propidium iodide. J Histochem Cytochem. 1985;33:77-79.

Kampmann M, Stock D. Reverse gyrase has heat-protective DNA chaperone activity independent of supercoiling. Nucleic Acids Res. 2004;32:3537-3545.

Lipscomb GL, Hahn EM, Crowley AT, Adams MW. Reverse gyrase is essential for microbial growth at 95°C. Extremophiles. 2017;21:603-608.

López-García P. DNA supercoiling and temperature adaptation: a clue to early diversification of life?. J Mol Evol. 1999;49:439-452.

Mesle MM, Beam JP, Jay ZJ, Bodle B, Bogenschutz E, Inskeep WP. Hydrogen peroxide cycling in high-temperature acidic geothermal springs and potential implications for oxidative stress response. Front Mar Sci. 2017;4:130.

Mondal N, Roy C, Chatterjee S, Sarkar J, Dutta S, Bhattacharya S, et al. Thermal endurance by a hot-spring-dwelling phylogenetic relative of the mesophilic *Paracoccus*. Microbiol Spectr. 2022;10:e01606-22.

Mulkidjanian AY, Bychkov AY, Dibrova DV, Galperin MY, Koonin EV. Origin of first cells at terrestrial, anoxic geothermal fields. Proc Natl Acad Sci USA. 2012;109:E821-E830.

Murayama N, Shimizu H, Takiguchi S, Baba Y, Amino H, Horiuchi T, et al. Evidence for involvement of *Escherichia* *coli* genes pmbA, csrA and a previously unrecognized gene tldD, in the control of DNA gyrase by letD (ccdB) of sex factor F. J Mol Biol. 1996;256:483-502.

Page, R. and Peti, W., 2016. Toxin-antitoxin systems in bacterial growth arrest and persistence. Nat Chem Biol. 12, 208-214.

Quan X, Nitta K, Matoba O, Xia P, Awatsuji Y. Phase and fluorescence imaging by combination of digital holographic microscopy and fluorescence microscopy. Opt Rev. 2015 Apr;22:349-53.

Rai AP. Compilation of data on chemical analysis of water and gas samples from North West Himalaya and adjoining areas. Bulletin Series-C, No. 5, Geological Survey of India, 2001.

Roy C, Alam M, Mandal S, Haldar PK, Bhattacharya S, Mukherjee T, et al. Global association between thermophilicity and vancomycin susceptibility in bacteria. Front Microbiol. 2016;7:412.

Roy C, Mondal N, Peketi A, Fernandes S, Mapder T, Volvoikar SP, et al. Geomicrobial dynamics of Trans-Himalayan sulfur–borax spring system reveals mesophilic bacteria’s resilience to high heat. J Earth Syst Sci. 2020a;129:157.

Roy C, Rameez MJ, Haldar PK, Peketi A, Mondal N, Bakshi U, et al. Microbiome and ecology of a hot spring-microbialite system on the Trans-Himalayan Plateau. Sci Rep. 2020b;10:5917.

Sengupta S, Nagaraja V. YacG from *Escherichia* *coli* is a specific endogenous inhibitor of DNA gyrase. Nucleic Acids Res. 2008;36:4310-4316.

Sherpa MT, Das S, Thakur N. Physicochemical analysis of hot water springs of Sikkim-Polok tatopani, borong tatopani and Reshi tatopani. Recent Res Sci Technol. 2013;5:63-67.

Van Kranendonk MJ, Baumgartner R, Djokic T, Ota T, Steller L, Garbe U, et al. Elements for the origin of life on land: a deep-time perspective from the Pilbara Craton of Western Australia. Astrobiology. 2021;21:39-59.

Vezzi A, Campanaro S, D'angelo M, Simonato F, Vitulo N, Lauro FM, et al. Life at depth: *Photobacterium* *profundum* genome sequence and expression analysis. Science. 2005;307:1459-1461.

**References used in Tables S4, S5 and S6**

Ahmed, I., Yokota, A., Yamazoe, A. and Fujiwara, T., 2007. Proposal of *Lysinibacillus* *boronitolerans* gen. nov. sp. nov., and transfer of *Bacillus* *fusiformis* to *Lysinibacillus* *fusiformis* comb. nov. and *Bacillus* *sphaericus* to *Lysinibacillus* *sphaericus* comb. nov. *International Journal of Systematic and Evolutionary Microbiology*, *57*, 1117-1125.

Aizawa, T., Vijarnsorn, P., Nakajima, M. and Sunairi, M., 2011. *Burkholderia* *bannensis* sp. nov., an acid-neutralizing bacterium isolated from torpedo grass (*Panicum* *repens*) growing in highly acidic swamps. *International Journal of Systematic and Evolutionary Microbiology*, *61*, 1645-1650.

Albuquerque, L., Polónia, A.R.M., Barroso, C., Froufe, H.J., Lage, O., Lobo-da-Cunha, A., Egas, C. and da Costa, M.S., 2018. *Raineya* *orbicola* gen. nov., sp. nov. a slightly thermophilic bacterium of the phylum Bacteroidetes and the description of Raineyaceae fam. nov. *International Journal of Systematic and Evolutionary Microbiology*, *68*, 982-989.

Albuquerque, L., Rainey, F.A., Nobre, M.F. and da Costa, M.S., 2011. *Schleiferia* *thermophila* gen. nov., sp. nov., a slightly thermophilic bacterium of the phylum ‘Bacteroidetes’ and the proposal of Schleiferiaceae fam. nov. *International Journal of Systematic and Evolutionary Microbiology*, *61*, 2450-2455.

Albuquerque, L., Rainey, F.A., Nobre, M.F. and da Costa, M.S., 2008. *Elioraea* *tepidiphila* gen. nov., sp. nov., a slightly thermophilic member of the Alphaproteobacteria. *International Journal of Systematic and Evolutionary Microbiology*, *58*, 773-778.

Albuquerque, L., Simoes, C., Nobre, M.F., Pino, N.M., Battista, J.R., Silva, M.T., Rainey, F.A. and de Costa, M.S., 2005. *Truepera* *radiovictrix* gen. nov., sp. nov., a new radiation resistant species and the proposal of Trueperaceae fam. nov. *FEMS Microbiology Letters*, *247*, 161-169.

Amoozegar, M.A., Sánchez-Porro, C., Rohban, R., Hajighasemi, M. and Ventosa, A., 2009. *Piscibacillus* *halophilus* sp. nov., a moderately halophilic bacterium from a hypersaline Iranian lake. *International Journal of Systematic and Evolutionary Microbiology*, *59*, 3095-3099.

An, S.Y., Asahara, M., Goto, K., Kasai, H. and Yokota, A., 2007. *Terribacillus* *saccharophilus* gen. nov., sp. nov. and *Terribacillus* *halophilus* sp. nov., spore-forming bacteria isolated from field soil in Japan. *International Journal of Systematic and Evolutionary Microbiology*, *57*, 51-55.

Araújo, J., Flores-Félix, J.D., Igual, J.M., Peix, A., González-Andrés, F., Díaz-Alcántara, C.A. and Velázquez, E., 2017. *Bradyrhizobium* *cajani* sp. nov. isolated from nodules of *Cajanus* *cajan*. *International Journal of Systematic and Evolutionary Microbiology*, *67*, 2236-2241.

Arora, P.K., Chauhan, A., Pant, B., Korpole, S., Mayilraj, S. and Jain, R.K., 2011. *Chryseomicrobium* *imtechense* gen. nov., sp. nov., a new member of the family Planococcaceae. *International Journal of Systematic and Evolutionary Microbiology*, *61*, 1859-1864.

Asker, D., Beppu, T. and Ueda, K., 2008. *Nubsella* *zeaxanthinifaciens* gen. nov., sp. nov., a zeaxanthin-producing bacterium of the family Sphingobacteriaceae isolated from freshwater. *International Journal of Systematic and Evolutionary Microbiology*, *58*, 601-606.

Ben Aissa, F., Postec, A., Erauso, G., Payri, C., Pelletier, B., Hamdi, M., Fardeau, M.L. and Ollivier, B., 2015. Characterization of *Alkaliphilus* *hydrothermalis* sp. nov., a novel alkaliphilic anaerobic bacterium, isolated from a carbonaceous chimney of the Prony hydrothermal field, New Caledonia. *Extremophiles*, *19*, 183-188.

Bernard, K.A., Pacheco, A.L., Loomer, C., Burdz, T., Wiebe, D., Huynh, C., Kaplen, B., Olson, A.B., Cnockaert, M., Eguchi, H. and Kuwahara, T., 2016. *Corynebacterium* *lowii* sp. nov. and *Corynebacterium* *oculi* sp. nov., derived from human clinical disease and an emended description of *Corynebacterium* *mastitidis*. *International Journal of Systematic and Evolutionary Microbiology*, *66*, 2803-2812.

Bes, M., Merrouch, M., Joseph, M., Quemeneur, M., Payri, C., Pelletier, B., Ollivier, B., Fardeau, M.L., Erauso, G. and Postec, A., 2015. *Acetoanaerobium* *pronyense* sp. nov., an anaerobic alkaliphilic bacterium isolated from a carbonate chimney of the Prony Hydrothermal Field (New Caledonia). *International Journal of Systematic and Evolutionary Microbiology*, *65*, 2574-2580.

Boden, R., 2017. Reclassification of *Halothiobacillus* *hydrothermalis* and *Halothiobacillus* *halophilus* to *Guyparkeria* gen. nov. in the Thioalkalibacteraceae fam. nov., with emended descriptions of the genus *Halothiobacillus* and family Halothiobacillaceae. *International Journal of Systematic and Evolutionary Microbiology*, *67*, 3919-3928.

Boldareva, E.N., Akimov, V.N., Boychenko, V.A., Stadnichuk, I.N., Moskalenko, A.A., Makhneva, Z.K. and Gorlenko, V.M., 2008. *Rhodobaca* *barguzinensis* sp. nov., a new alkaliphilic purple nonsulfur bacterium isolated from a soda lake of the Barguzin Valley (Buryat Republic, Eastern Siberia). *Microbiology*, *77*, 206-218.

Bowman, J.P., Sly, L.I., Nichols, P.D. and Hayward, A.C., 1993. Revised taxonomy of the methanotrophs: description of *Methylobacter* gen. nov., emendation of *Methylococcus*, validation of *Methylosinus* and *Methylocystis* species, and a proposal that the family Methylococcaceae includes only the group I methanotrophs. *International Journal of Systematic and Evolutionary Microbiology*, *43*, 735-753.

Buddhi, S., Gupta, D., Ch, S. and Ch. V, R., 2020. *Afifella* *aestuarii* sp. nov., a phototrophic bacterium. *International Journal of Systematic and Evolutionary Microbiology*, *70*, 327-333.

Burger-Wiersma, T., Stal, L.J. and Mur, L.R., 1989. *Prochlorothrix* *hollandica* gen. nov., sp. nov., a filamentous oxygenic photoautotrophic procaryote containing chlorophylls a and b: assignment to Prochlorotrichaceae fam. nov. and order Prochlorales Florenzano, Balloni, and Materassi 1986, with emendation of the ordinal description. *International Journal of Systematic and Evolutionary Microbiology*, *39*, 250-257.

Cai, J., Wang, Y., Liu, D., Zeng, Y., Xue, Y., Ma, Y. and Feng, Y., 2007. *Fervidobacterium* *changbaicum* sp. nov., a novel thermophilic anaerobic bacterium isolated from a hot spring of the Changbai Mountains, China. *International Journal of Systematic and Evolutionary Microbiology*, *57*, 2333-2336.

Casida Jr, L.E., 1982. *Ensifer* *adhaerens* gen. nov., sp. nov.: a bacterial predator of bacteria in soil. *International Journal of Systematic and Evolutionary Microbiology*, *32*, 339-345.

Castellano-Hinojosa, A., Correa-Galeote, D., Ramírez-Bahena, M.H., Tortosa, G., González-López, J., Bedmar, E.J. and Peix, Á., 2021. *Agrobacterium* *leguminum* sp. nov., isolated from nodules of *Phaseolus* *vulgaris* in Spain. *International Journal of Systematic and Evolutionary Microbiology*, *71*, 005120.

Chang, Y.H., Han, J.I., Chun, J., Lee, K.C., Rhee, M.S., Kim, Y.B. and Bae, K.S., 2002. *Comamonas* *koreensis* sp. nov., a non-motile species from wetland in Woopo, Korea. *International Journal of Systematic and Evolutionary Microbiology*, *52*, 377-381.

Chen, M.H., Sheu, S.Y., James, E.K., Young, C.C. and Chen, W.M., 2013a. *Azoarcus olearius* sp. nov., a nitrogen-fixing bacterium isolated from oil-contaminated soil. *International Journal of Systematic and Evolutionary Microbiology*, *63*, 3755-3761.

Chen, W.M., Huang, H.W., Chang, J.S., Han, Y.L., Guo, T.R. and Sheu, S.Y., 2013b. *Tepidimonas* *fonticaldi* sp. nov., a slightly thermophilic betaproteobacterium isolated from a hot spring. *International Journal of Systematic and Evolutionary Microbiology*, *63*, 1810-1816.

Chen, W.M., Lin, Y.S., Sheu, D.S. and Sheu, S.Y., 2012. *Delftia* *litopenaei* sp. nov., a poly-β-hydroxybutyrate-accumulating bacterium isolated from a freshwater shrimp culture pond. *International Journal of Systematic and Evolutionary Microbiology*, *62*, 2315-2321.

Chen, Y.G., Zhang, Y.Q., Shi, J.X., Xiao, H.D., Tang, S.K., Liu, Z.X., Huang, K., Cui, X.L. and Li, W.J., 2009. *Jeotgalicoccus* *marinus* sp. nov., a marine bacterium isolated from a sea urchin. *International Journal of Systematic and Evolutionary Microbiology*, *59*, 1625-1629.

Chern, L.L., Stackebrandt, E., Lee, S.F., Lee, F.L., Chen, J.K. and Fu, H.M., 2004. *Chitinibacter* *tainanensis* gen. nov., sp. nov., a chitin-degrading aerobe from soil in Taiwan. *International Journal of Systematic and Evolutionary Microbiology*, *54*, 1387-1391.

Chiu, H.H., Rogozin, D.Y., Huang, S.P., Degermendzhy, A.G., Shieh, W.Y. and Tang, S.L., 2014. *Aliidiomarina* *shirensis* sp. nov., a halophilic bacterium isolated from Shira Lake in Khakasia, southern Siberia, and a proposal to transfer *Idiomarina* *maris* to the genus *Aliidiomarina*. *International Journal of Systematic and Evolutionary Microbiology*, *64*, 1334-1339.

Choi, J.H., Kim, M.S., Roh, S.W. and Bae, J.W., 2010. *Brevundimonas* *basaltis* sp. nov., isolated from black sand. *International Journal of Systematic and Evolutionary Microbiology*, *60*, 1488-1492.

Chun, B.H., Lee, Y., Jin, H.M. and Jeon, C.O., 2017. *Cloacibacterium* *caeni* sp. nov., isolated from activated sludge. *International Journal of Systematic and Evolutionary Microbiology*, *67*, 1688-1692.

Chung, A.P., Rainey, F.A., Valente, M., Nobre, M.F. and da Costa, M.S., 2000. *Thermus* *igniterrae* sp. nov. and *Thermus* *antranikianii* sp. nov., two new species from Iceland. *International Journal of Systematic and Evolutionary Microbiology*, *50*, 209-217.

Cihan, A.C., Cokmus, C., Koc, M. and Ozcan, B., 2014. *Anoxybacillus* *calidus* sp. nov., a thermophilic bacterium isolated from soil near a thermal power plant. *International Journal of Systematic and Evolutionary Microbiology*, *64*, 211-219

Collins, M.D. and Lawson, P.A., 2000. The genus *Abiotrophia* (Kawamura et al.) is not monophyletic: proposal of *Granulicatella* gen. nov., *Granulicatella* *adiacens* comb. nov., *Granulicatella* *elegans* comb. nov. and *Granulicatella* *balaenopterae* comb. nov. *International Journal of Systematic and Evolutionary Microbiology*, *50*, 365-369.

Copeland, J.J., 1936. Yellowstone thermal myxophyceae. *Annals of the New York Academy of Sciences*, *36*, 4-223.

Crapart, S., Fardeau, M.L., Cayol, J.L., Thomas, P., Sery, C., Ollivier, B. and Combet-Blanc, Y., 2007. *Exiguobacterium* *profundum* sp. nov., a moderately thermophilic, lactic acid-producing bacterium isolated from a deep-sea hydrothermal vent. *International Journal of Systematic and Evolutionary Microbiology*, *57*, 287-292.

De la Fuente, R., Suarez, G. and Schleifer, K.H., 1985. *Staphylococcus* *aureus* subsp. anaerobius subsp. nov., the causal agent of abscess disease of sheep. *International Journal of Systematic Bacteriology*, *35*, 99-102.

Dekio, I., Sakamoto, M., Suzuki, T., Yuki, M., Kinoshita, S., Murakami, Y. and Ohkuma, M., 2020. *Cutibacterium* *modestum* sp. nov., isolated from meibum of human meibomian glands, and emended descriptions of *Cutibacterium* *granulosum* and *Cutibacterium* *namnetense*. *International Journal of Systematic and Evolutionary Microbiology*, *70*, 2457-2462.

Dridi, B., Fardeau, M.L., Ollivier, B., Raoult, D. and Drancourt, M., 2012. *Methanomassiliicoccus* *luminyensis* gen. nov., sp. nov., a methanogenic archaeon isolated from human faeces. *International Journal of Systematic and Evolutionary Microbiology*, *62*, 1902-1907.

Duan, L., Li, J.L., Li, X., Dong, L., Fang, B.Z., Xiao, M., Mou, X. and Li, W.J., 2020. *Roseibium* *aestuarii* sp. nov., isolated from Pearl River Estuary. *International Journal of Systematic and Evolutionary Microbiology*, *70*, 2896-2900.

Emborg, J., Dalgaard, P. and Ahrens, P., 2006. *Morganella* *psychrotolerans* sp. nov., a histamine-producing bacterium isolated from various seafoods. *International Journal of Systematic and Evolutionary Microbiology*, *56*, 2473-2479.

Engle, M., Li, Y., Woese, C. and Wiegel, J., 1995. Isolation and Characterization of a Novel Alkalitolerant Thermophile, *Anaerobranca* *horikoshii* gen. nov., sp. nov. *International Journal of Systematic and Evolutionary Microbiology*, *45*, 454-461.

Faine, S. and Stallman, N.D., 1982. Amended descriptions of the genus *Leptospira* *Noguchi* 1917 and the species *L*. *interrogans* (Stimson 1907) Wenyon 1926 and *L*. *biflexa* (Wolbach and Binger 1914) Noguchi 1918. *International Journal of Systematic and Evolutionary Microbiology*, *32*, 461-463.

Falsen, E., Collins, M.D., Welinder-Olsson, C., Song, Y., Finegold, S.M. and Lawson, P.A., 2005. *Fastidiosipila* *sanguinis* gen. nov., sp. nov., a new Gram-positive, coccus-shaped organism from human blood. *International Journal of Systematic and Evolutionary Microbiology*, *55*, 853-858.

Fan, M.C., Guo, Y.Q., Zhang, L.P., Zhu, Y.M., Chen, W.M., Lin, Y.B. and Wei, G.H., 2018. *Herbaspirillum* *robiniae* sp. nov., isolated from root nodules of *Robinia* *pseudoacacia* in a lead–zinc mine. *International Journal of Systematic and Evolutionary Microbiology*, *68*, 1300-1306.

Fan, Y., Jin, Z., Tong, J., Li, W., Pasciak, M., Gamian, A., Liu, Z. and Huang, Y., 2002. *Rothia* *amarae* sp. nov., from sludge of a foul water sewer. *International Journal of Systematic and Evolutionary Microbiology*, *52*, 2257-2260.

Felföldi, T., Kéki, Z., Sipos, R., Márialigeti, K., Tindall, B.J., Schumann, P. and Tóth, E.M., 2011. *Ottowia* *pentelensis* sp. nov., a floc-forming betaproteobacterium isolated from an activated sludge system treating coke plant effluent. *International Journal of Systematic and Evolutionary Microbiology*, *61*, 2146-2150.

Foster, G., Osterman, B.S., Godfroid, J., Jacques, I. and Cloeckaert, A., 2007. *Brucella* *ceti* sp. nov. and *Brucella* *pinnipedialis* sp. nov. for *Brucella* strains with cetaceans and seals as their preferred hosts. *International Journal of Systematic and Evolutionary Microbiology*, *57*, 2688-2693.

Francis, I.M., Jochimsen, K.N., De Vos, P. and van Bruggen, A.H., 2014. Reclassification of rhizosphere bacteria including strains causing corky root of lettuce and proposal of *Rhizorhapis* *suberifaciens* gen. nov., comb. nov., *Sphingobium* *mellinum* sp. nov., *Sphingobium* *xanthum* sp. nov. and *Rhizorhabdus* *argentea* gen. nov., sp. nov. *International Journal of Systematic and Evolutionary Microbiology*, *64*, 1340-1350.

Gaisin, V.A., Kalashnikov, A.M., Grouzdev, D.S., Sukhacheva, M.V., Kuznetsov, B.B. and Gorlenko, V.M., 2017. *Chloroflexus* *islandicus* sp. nov., a thermophilic filamentous anoxygenic phototrophic bacterium from a geyser. *International Journal of Systematic and Evolutionary Microbiology*, *67*, 1381-1386.

Gao, J.L., Sun, P., Wang, X.M., Cheng, S., Lv, F., Qiu, T.L., Yuan, M. and Sun, J.G., 2016. *Sphingomonaszeicaulis* sp. nov., an endophytic bacterium isolated from maize root. *International Journal of Systematic and Evolutionary Microbiology*, *66*, 3755-3760.

Geiger, A., Fardeau, M.L., Falsen, E., Ollivier, B. and Cuny, G., 2010. *Serratia* *glossinae* sp. nov., isolated from the midgut of the tsetse fly *Glossina* *palpalis* *gambiensis*. *International Journal of Systematic and Evolutionary Microbiology*, *60*, 1261-1265.

Gomila, M., Bowien, B., Falsen, E., Moore, E.R. and Lalucat, J., 2007. Description of *Pelomonas* *aquatica* sp. nov. and *Pelomonas* *puraquae* sp. nov., isolated from industrial and haemodialysis water. *International Journal of Systematic and Evolutionary Microbiology*, *57*, 2629-2635.

Götz, D., Banta, A., Beveridge, T.J., Rushdi, A.I., Simoneit, B.R.T. and Reysenbach, A.L., 2002. *Persephonella* *marina* gen. nov., sp. nov. and *Persephonella* *guaymasensis* sp. nov., two novel, thermophilic, hydrogen-oxidizing microaerophiles from deep-sea hydrothermal vents. *International Journal of Systematic and Evolutionary Microbiology*, *52*, 1349-1359.

Green, P.N. and Ardley, J.K., 2018. Review of the genus *Methylobacterium* and closely related organisms: a proposal that some *Methylobacterium* species be reclassified into a new genus, *Methylorubrum* gen. nov. *International Journal of Systematic and Evolutionary Microbiology*, *68*, 2727-2748.

Groth, I., Schumann, P., Weiss, N., Schuetze, B., Augsten, K. and Stackebrandt, E., 2001. *Ornithinimicrobium* *humiphilum* gen. nov., sp. nov., a novel soil actinomycete with L-ornithine in the peptidoglycan. *International Journal of Systematic and Evolutionary Microbiology*, *51*, 81-87.

Guan, T.W., Xiao, J., Zhao, K., Luo, X.X., Zhang, X.P. and Zhang, L.L., 2010. Halomonas xinjiangensis sp. nov., a halotolerant bacterium isolated from a salt lake. *International Journal of Systematic and Evolutionary Microbiology*, *60*, 349-352.

Gupta, R.S., Patel, S., Saini, N. and Chen, S., 2020. Robust demarcation of 17 distinct *Bacillus* species clades, proposed as novel Bacillaceae genera, by phylogenomics and comparative genomic analyses: description of *Robertmurraya* *kyonggiensis* sp. nov. and proposal for an emended genus *Bacillus* *limiting* it only to the members of the *Subtilis* and *Cereus* clades of species. *International Journal of Systematic and Evolutionary Microbiology*, *70*, 5753-5798.

Hetzer, A., McDonald, I.R. and Morgan, H.W., 2008. *Venenivibrio* *stagnispumantis* gen. nov., sp. nov., a thermophilic hydrogen-oxidizing bacterium isolated from Champagne Pool, Waiotapu, New Zealand. *International Journal of Systematic and Evolutionary Microbiology*, *58*, 398-403.

Heylen, K., Lebbe, L. and De Vos, P., 2008. *Acidovorax* *caeni* sp. nov., a denitrifying species with genetically diverse isolates from activated sludge. *International Journal of Systematic and Evolutionary Microbiology*, *58*, 73-77.

Holt, J.G., 1984. Bergey’s Manual of Determinative Bacteriology, Vol. 1. *Williams and Wilkins, Baltimore*.

Huber, R., Eder, W., Heldwein, S., Wanner, G., Huber, H., Rachel, R. and Stetter, K.O., 1998. *Thermocrinis* *ruber* gen. nov., sp. nov., a pink-filament-forming hyperthermophilic bacterium isolated from Yellowstone National Park. *Applied and Environmental Microbiology*, *64*, 3576-3583.

Huber, R., Kristjansson, J.K. and Stetter, K.O., 1987. *Pyrobaculum* gen. nov., a new genus of neutrophilic, rod-shaped archaebacteria from continental solfataras growing optimally at 100°C. *Archives of Microbiology*, *149*, 95-101.

Huber, R., Wilharm, T., Huber, D., Trincone, A., Burggraf, S., König, H., Reinhard, R., Rockinger, I., Fricke, H. and Stetter, K.O., 1992. *Aquifex* *pyrophilus* gen. nov. sp. nov., represents a novel group of marine hyperthermophilic hydrogen-oxidizing bacteria. *Systematic and Applied Microbiology*, *15*, 340-351.

Huys, G., Kämpfer, P., Albert, M.J., Kühn, I., Denys, R. and Swings, J., 2002. *Aeromonas* *hydrophila* subsp. *dhakensis* subsp. nov., isolated from children with diarrhoea in Bangladesh, and extended description of *Aeromonas* *hydrophila* subsp. *hydrophila* (Chester 1901) Stanier 1943 (approved lists 1980). *International Journal of Systematic and Evolutionary Microbiology*, *52*, 705-712.

Iino, T., Mori, K. and Suzuki, K.I., 2010. *Methanospirillum* *lacunae* sp. nov., a methane-producing archaeon isolated from a puddly soil, and emended descriptions of the genus *Methanospirillum* and *Methanospirillum* *hungatei*. *International Journal of Systematic and Evolutionary Microbiology*, *60*, 2563-2566.

Ishikawa, M., Nakajima, K., Itamiya, Y., Furukawa, S., Yamamoto, Y. and Yamasato, K., 2005. *Halolactibacillus* *halophilus* gen. nov., sp. nov. and *Halolactibacillus* *miurensis* sp. nov., halophilic and alkaliphilic marine lactic acid bacteria constituting a phylogenetic lineage in *Bacillus* rRNA group 1. *International Journal of Systematic and Evolutionary Microbiology*, *55*, 2427-2439.

Ito, T., Sugita, K., Yumoto, I., Nodasaka, Y. and Okabe, S., 2005. *Thiovirga* *sulfuroxydans* gen. nov., sp. nov., a chemolithoautotrophic sulfur-oxidizing bacterium isolated from a microaerobic waste-water biofilm. *International Journal of Systematic and Evolutionary Microbiology*, *55*, 1059-1064.

Jayasinghearachchi, H.S. and Lal, B., 2011. *Oceanotoga* *teriensis* gen. nov., sp. nov., a thermophilic bacterium isolated from offshore oil-producing wells. *International Journal of Systematic and EvolutionaryMicrobiology*, *61*, 554-560.

Ji, S., Zhao, R., Li, Z., Li, B., Shi, X. and Zhang, X.H., 2013. *Ferrimonas* *sediminum* sp. nov., isolated from coastal sediment of an amphioxus breeding zone. *International Journal of Systematic and Evolutionary Microbiology*, *63*, 977-981.

Jin, L., Lee, H.G., Kim, H.S., Ahn, C.Y. and Oh, H.M., 2013. *Caulobacter* *daechungensis* sp. nov., a stalked bacterium isolated from a eutrophic reservoir. *International Journal of Systematic and Evolutionary Microbiology*, *63*, 2559-2564.

Jochimsen, B., Peinemann-Simon, S., Völker, H., Stüben, D., Botz, R., Stoffers, P., Dando, P.R. and Thomm, M., 1997. *Stetteria* *hydrogenophila*, gen. nov. and sp. nov., a novel mixotrophic sulfur-dependent crenarchaeote isolated from Milos, Greece. *Extremophiles*, *1*, 67-73.

Kämpfer, P., Arun, A.B., Frischmann, A., Busse, H.J., Young, C.C., Rekha, P.D. and Chen, W.M., 2013. *Stappia* *taiwanensis* sp. nov., isolated from a coastal thermal spring. *International Journal of Systematic and Evolutionary Microbiology*, *63*, 1350-1354.

Kämpfer, P., Matthews, H., Glaeser, S.P., Martin, K., Lodders, N. and Faye, I., 2011. *Elizabethkingia* *anophelis* sp. nov., isolated from the midgut of the mosquito *Anopheles* *gambiae*. *International Journal of Systematic and Evolutionary Microbiology*, *61*, 2670-2675.

Kashefi, K., Holmes, D.E., Reysenbach, A.L. and Lovley, D.R., 2002. Use of Fe (III) as an electron acceptor to recover previously uncultured hyperthermophiles: isolation and characterization of *Geothermobacterium* *ferrireducens* gen. nov., sp. nov. *Applied and Environmental Microbiology*, *68*, 1735-1742.

Khan, I.U., Habib, N., Xiao, M., Huang, X., Khan, N.U., Im, W.T., Ahmed, I., Zhi, X.Y. and Li, W.J., 2018. *Phenylobacterium* *terrae* sp. nov., isolated from a soil sample of Khyber-Pakhtun-Khwa, Pakistan. *Antonie Van Leeuwenhoek*, *111*, 1767-1775.

Kilpper-Bälz, R. and Schleifer, K.H., 1988. Transfer of *Streptococcus* *morbillorum* to the Genus *Gemella* as *Gemella* *morbillorum* comb. nov. *International Journal of Systematic and Evolutionary Microbiology*, *38*, 442-443.

Kim, J., Srinivasan, S., You, T., Bang, J.J., Park, S. and Lee, S.S., 2013. *Brevibacterium* *ammoniilyticum* sp. nov., an ammonia-degrading bacterium isolated from sludge of a wastewater treatment plant. *International Journal of Systematic and Evolutionary Microbiology*, *63*, 1111-1118.

Kim, M.K., Kim, T.S., Joung, Y., Han, J.H. and Kim, S.B., 2016. *Taibaiella* *soli* sp. nov., isolated from pine forest soil. *International Journal of Systematic and Evolutionary Microbiology*, *66*, 3230-3234.

Kim, S.B. and Goodfellow, M., 1999. Reclassification of *Amycolatopsis* *rugosa* as *Prauserella* *rugosa* gen. nov., comb. nov. *International Journal of Systematic and Evolutionary Microbiology*, *49*, 507-512.

Koh, H.W., Hong, H., Min, U.G., Kang, M.S., Kim, S.G., Na, J.G., Rhee, S.K. and Park, S.J., 2015. *Rhodanobacter* *aciditrophus* sp. nov., an acidophilic bacterium isolated from mine wastewater. *International Journal of Systematic and Evolutionary Microbiology*, *65*, 4574-4579.

Komárek, J., Johansen, J.R., Šmarda, J. and Strunecký, O., 2020. Phylogeny and taxonomy of *Synechococcus*-like cyanobacteria. *Fottea*, *20*, 171-191.

Koval, S.F., Hynes, S.H., Flannagan, R.S., Pasternak, Z., Davidov, Y. and Jurkevitch, E., 2013. *Bdellovibrio* *exovorus* sp. nov., a novel predator of *Caulobacter* *crescentus*. *International Journal of Systematic and Evolutionary Microbiology*, *63*, 146-151.

Kumari, H., Gupta, S.K., Jindal, S., Katoch, P. and Lal, R., 2009. *Sphingobium* *lactosutens* sp. nov., isolated from a hexachlorocyclohexane dump site and *Sphingobium* *abikonense* sp. nov., isolated from oil-contaminated soil. *International Journal of Systematic and Evolutionary Microbiology*, *59*, 2291-2296.

Kumari, P., Bhattacharjee, S., Poddar, A. and Das, S.K., 2016. *Sulfitobacter* faviae sp. nov., isolated from the coral *Faviaveroni*. *International Journal of Systematic and Evolutionary Microbiology*, *66*, 3786-3792.

Kusunoki, S. and Ezaki, T., 1992. Proposal of *Mycobacterium* *peregrinum* sp. nov., nom. rev., and Elevation of *Mycobacterium* *chelonae* subsp. *abscessus* (Kubica et al.) to Species Status: *Mycobacterium* *abscessus* comb. nov. *International Journal of Systematic and Evolutionary Microbiology*, *42*, 240-245.

Lau, S.C., Tsoi, M.M., Li, X., Dobretsov, S., Plakhotnikova, Y., Wong, P.K. and Qian, P.Y., 2005. *Pseudoalteromonas* *spongiae* sp. nov., a novel member of the γ-Proteobacteria isolated from the sponge *Mycale* *adhaerens* in Hong Kong waters. *International Journal of Systematic and Evolutionary Microbiology*, *55*, 1593-1596.

Lee, E.M., Jeon, C.O., Choi, I., Chang, K.S. and Kim, C.J., 2005. *Silanimonas* *lenta* gen. nov., sp. nov., a slightly thermophilic and alkaliphilic gammaproteobacterium isolated from a hot spring. *International Journal of Systematic and Evolutionary Microbiology*, *55*, 385-389.

Lee, J.C., Kim, S.G. and Whang, K.S., 2014. *Novosphingobium* *aquiterrae* sp. nov., isolated from ground water. *International Journal of Systematic and Evolutionary Microbiology*, *64*, 3282-3287.

Lee, J.C., Kim, Y.S., Yun, B.S. and Whang, K.S., 2015. *Idiomarina* *halophila* sp. nov., isolated from a solar saltern sediment. *International Journal of Systematic and Evolutionary Microbiology*, *65*, 1268-1273.

Lee, M., Woo, S.G., Chae, M., Shin, M.C., Jung, H.M. and Ten, L.N., 2011. *Stenotrophomonas* *daejeonensis* sp. nov., isolated from sewage. *International Journal of Systematic and Evolutionary Microbiology*, *61*, 598-604.

Lee, Y.J., Romanek, C.S., Mills, G.L., Davis, R.C., Whitman, W.B. and Wiegel, J., 2006. *Gracilibacter* *thermotolerans* gen. nov., sp. nov., an anaerobic, thermotolerant bacterium from a constructed wetland receiving acid sulfate water. *International Journal of Systematic and Evolutionary Microbiology*, *56*, 2089-2093.

Liu, S., Jin, D., Lan, R., Wang, Y., Meng, Q., Dai, H., Lu, S., Hu, S. and Xu, J., 2015. *Escherichia* *marmotae* sp. nov., isolated from faeces of Marmota himalayana. *International Journal of Systematic and Evolutionary Microbiology*, *65*, 2130-2134.

Lu, C.Y., Li, Y.Q., Tian, Y., Han, M.X., Rao, M.P.N., Li, Y.R., Zhu, Z.N., Wei, D.Q., An, D.D. and Li, W.J., 2017. *Alcaligenes* *endophyticus* sp. nov., isolated from roots of *Ammodendron* *bifolium*. *International Journal of Systematic and Evolutionary Microbiology*, *67*, 939-943.

MacDonell, M.T. and Colwell, R.R., 1985. Phylogeny of the Vibrionaceae, and recommendation for two new genera, *Listonella* and *Shewanella*. *Systematic and Applied Microbiology*, *6*, 171-182.

Maruyama, A., Honda, D., Yamamoto, H., Kitamura, K. and Higashihara, T., 2000. Phylogenetic analysis of psychrophilic bacteria isolated from the Japan Trench, including a description of the deep-sea species *Psychrobacter* *pacificensis* sp. nov. *International Journal of Systematic and Evolutionary Microbiology*, *50*, 835-846.

Mediannikov, O., Sekeyová, Z., Birg, M.L. and Raoult, D., 2010. A novel obligate intracellular gamma-proteobacterium associated with ixodid ticks, *Diplorickettsia* *massiliensis*, Gen. Nov., Sp. Nov. *PloS One*, *5*, e11478.

Mehnaz, S., Weselowski, B. and Lazarovits, G., 2007. *Azospirillum* *canadense* sp. nov., a nitrogen-fixing bacterium isolated from corn rhizosphere. *International Journal of Systematic and Evolutionary Microbiology*, *57*, 620-624.

Mellado, E., Moore, E.R.B., Nieto, J.J. and Ventosa, A., 1996. Analysis of 16S rRNA gene sequences of *Vibrio* *costicola* strains: description of *Salinivibrio* *costicola* gen. nov., comb. nov. *International Journal of Systematic Bacteriology*, *46*, 817-821.

Meng, Y.C., Liu, H.C., Zhou, Y.G., Cai, M. and Kang, Y., 2018. *Vibrio* *gangliei* sp. nov., a novel member of Vibrionaceae isolated from sawdust in a pigpen. *International Journal of Systematic and Evolutionary Microbiology*, *68*, 1969-1974.

Miroshnichenko, M.L., Lebedinsky, A.V., Chernyh, N.A., Tourova, T.P., Kolganova, T.V., Spring, S. and Bonch-Osmolovskaya, E.A., 2009. *Caldimicrobium* *rimae* gen. nov., sp. nov., an extremely thermophilic, facultatively lithoautotrophic, anaerobic bacterium from the Uzon Caldera, Kamchatka. *International Journal of Systematic and Evolutionary Microbiology*, *59*, 1040-1044.

Morales Nicolàs, G., Llorente i Cabratosa, I., Montesinos Seguí, E. and Moragrega i Garcia, C., 2017. A model for predicting *Xanthomonas* *arboricola* pv. pruni growth as a function of temperature. *PLoS One,5, e0177583*.

Mori, K. and Suzuki, K.I., 2008. *Thiofaba* *tepidiphila* gen. nov., sp. nov., a novel obligately chemolithoautotrophic, sulfur-oxidizing bacterium of the Gammaproteobacteria isolated from a hot spring. *International Journal of Systematic and Evolutionary Microbiology*, *58*, 1885-1891.

Müller, H.E., Brenner, D.J., Fanning, G.R., Grimont, P.A. and Kämpfer, P., 1996. Emended description of *Buttiauxella* *agrestis* with recognition of six new species of *Buttiauxella* and two new species of *Kluyvera*: *Buttiauxella* *ferragutiae* sp. nov., *Buttiauxella* *gaviniae* sp. nov., *Buttiauxella* *brennerae* sp. nov., *Buttiauxella* *izardii* sp. nov., *Buttiauxella* *noackiae* sp. nov., *Buttiauxella* *warmboldiae* sp. nov., *Kluyvera* *cochleae* sp. nov., and *Kluyvera* *georgiana* sp. nov. *International Journal of Systematic Bacteriology*, *46*, 50-63.

Nie, G.X., Ming, H., Li, S., Zhou, E.M., Cheng, J., Yu, T.T., Zhang, J., Feng, H.G., Tang, S.K. and Li, W.J., 2012. *Geodermatophilus* *nigrescens* sp. nov., isolated from a dry-hot valley. *Antonie Van Leeuwenhoek*, *101*, 811-817.

Ntougias, S. and Russell, N.J., 2001. *Alkalibacterium* *olivoapovliticus* gen. nov., sp. nov., a new obligately alkaliphilic bacterium isolated from edible-olive wash-waters. *International Journal of Systematic and Evolutionary Microbiology*, *51*, 1161-1170.

Ogg, C.D. and Patel, B.K., 2009. *Thermotalea* *metallivorans* gen. nov., sp. nov., a thermophilic, anaerobic bacterium from the Great Artesian Basin of Australia aquifer. *International Journal of Systematic and Evolutionary Microbiology*, *59*, 964-971.

O'Neill, A.H., Liu, Y., Ferrera, I., Beveridge, T.J. and Reysenbach, A.L., 2008. *Sulfurihydrogenibium* *rodmanii* sp. nov., a sulfur-oxidizing chemolithoautotroph from the Uzon Caldera, Kamchatka Peninsula, Russia, and emended description of the genus *Sulfurihydrogenibium*. *International Journal of Systematic and Evolutionary Microbiology*, 58, 1147-1152.

Page, R. and Peti, W., 2016. Toxin-antitoxin systems in bacterial growth arrest and persistence. *Nature Chemical Biology*, *12*, 208-214.

Palmer, A., Painter, J., Hassler, H., Richards, V.P., Bruce, T., Morrison, S., Brown, E., Kozak‐Muiznieks, N.A., Lucas, C. and McNealy, T.L., 2016. *Legionella* *clemsonensis* sp. nov.: a green fluorescing *Legionella* strain from a patient with pneumonia. *Microbiology and Immunology*, *60*, 694-701.

Patel, S. and Gupta, R.S., 2020. A phylogenomic and comparative genomic framework for resolving the polyphyly of the genus *Bacillus*: Proposal for six new genera of *Bacillus* species, *Peribacillus* gen. nov., *Cytobacillus* gen. nov., *Mesobacillus* gen. nov., *Neobacillus* gen. nov., *Metabacillus* gen. nov. and *Alkalihalobacillus* gen. nov. *International Journal of Systematic and Evolutionary Microbiology*, *70*, 406-438.

Pérez-Cataluña, A., Salas-Massó, N., Diéguez, A.L., Balboa, S., Lema, A., Romalde, J.L. and Figueras, M.J., 2019. Corrigendum (2): revisiting the taxonomy of the genus *Arcobacter*: getting order from the chaos. *Frontiers in Microbiology*, *10*, 2253.

Prauser, H., 1976. *Nocardioides*, a new genus of the order Actinomycetales. *International Journal of Systematic and Evolutionary Microbiology*, *26*, 58-65.

Rani, A., Sharma, A., Adak, T. and Bhatnagar, R.K., 2010. *Pseudoxanthomonas* *icgebensis* sp. nov., isolated from the midgut of *Anopheles* *stephensi* field-collected larvae. *The Journal of Microbiology*, *48*, 601-606.

Ren, B., Yang, N., Wang, J., Ma, X.L., Wang, Q., Xie, F., Guo, H., Liu, Z.H., Pugin, B. and Zhang, L.X., 2013. *Amphibacillus* *marinus* sp. nov., a member of the genus *Amphibacillus* isolated from marine mud. *International Journal of Systematic and Evolutionary Microbiology*, *63*, 1485-1491.

Romanenko, L.A., Tanaka, N., Frolova, G.M. and Mikhailov, V.V., 2008. *Pseudochrobactrum* *glaciei* sp. nov., isolated from sea ice collected from Peter the Great Bay of the Sea of Japan. *International Journal of Systematic and Evolutionary Microbiology*, *58*, 2454-2458.

Ryu, S.H., Chung, B.S., Le, N.T., Jang, H.H., Yun, P.Y., Park, W. and Jeon, C.O., 2008. *Devosi ageojensis* sp. nov., isolated from diesel-contaminated soil in Korea. *International Journal of Systematic and Evolutionary Microbiology*, *58*, 633-636.

Ryu, S.H., Chung, B.S., Park, M., Lee, S.S., Lee, S.S. and Jeon, C.O., 2008. *Rheinheimera* *soli* sp. nov., a gammaproteobacterium isolated from soil in Korea. *International Journal of Systematic and Evolutionary Microbiology*, *58*, 2271-2274.

Saiki, T., Kobayashi, Y., Kawagoe, K. and Beppu, T., 1985. *Dictyoglomus* *thermophilum* gen. nov., sp. nov., a chemoorganotrophic, anaerobic, thermophilic bacterium. *International Journal of Systematic and Evolutionary Microbiology*, *35*, 253-259.

Sako, Y., Nomura, N., Uchida, A., Ishida, Y., Morii, H., Koga, Y., Hoaki, T. and Maruyama, T., 1996. *Aeropyrum* *pernix* gen. nov., sp. nov., a novel aerobic hyperthermophilic archaeon growing at temperatures up to 100°C. *International Journal of Systematic Bacteriology*, *46*, 1070-1077.

Schleifer, K.H. and Kilpper-Bälz, R., 1984. Transfer of *Streptococcus* *faecalis* and *Streptococcus* *faecium* to the genus *Enterococcus* nom. rev. as *Enterococcus* *faecalis* comb. nov. and *Enterococcus* *faecium* comb. nov. *International Journal of Systematic Bacteriology*, *34*, 31-34.

Schleifer, K.H., Kraus, J., Dvorak, C., Kilpper-Bälz, R., Collins, M.D. and Fischer, W., 1985. Transfer of *Streptococcus* *lactis* and related streptococci to the genus *Lactococcus* gen. nov. *Systematic and Applied Microbiology*, 6, 183-195.

Shah, H.N. and Collins, D.M., 1990. *Prevotella*, a new genus to include *Bacteroides* *melaninogenicus* and related species formerly classified in the genus *Bacteroides*. *International Journal of Systematic and Evolutionary Microbiology*, *40*, 205-208.

Shah, H.N. and Collins, M.D., 1988. Proposal for reclassification of *Bacteroides* *asaccharolyticus*, *Bacteroides* *gingivalis*, and *Bacteroides* *endodontalis* in a new genus, *Porphyromonas*. *International Journal of Systematic and Evolutionary Microbiology*, *38*, 128-131.

Sherman, J.M. and Stark, P., 1931. *Streptococci* which grow at high temperatures. *Journal of Bacteriology*, *22*, 275.

Smet, A., Cools, P., Krizova, L., Maixnerova, M., Sedo, O., Haesebrouck, F., Kempf, M., Nemec, A. and Vaneechoutte, M., 2014. *Acinetobacter* *gandensis* sp. nov. isolated from horse and cattle. *International Journal of Systematic and Evolutionary Microbiology*, *64*, 4007-4015.

Sorokin, D.Y., 1992. *Catenococcus* *thiocyclus* gen. nov. sp. nov. - a new facultatively anaerobic bacterium from a near-shore sulphidic hydrothermal area. *Microbiology*, *138*, 2287-2292.

Sorokin, D.Y., Tourova, T.P., Spiridonova, E.M., Rainey, F.A. and Muyzer, G., 2005. *Thioclava* *pacifica* gen. nov., sp. nov., a novel facultatively autotrophic, marine, sulfur-oxidizing bacterium from a near-shore sulfidic hydrothermal area. *International Journal of Systematic and Evolutionary Microbiology*, *55*, 1069-1075.

Spring, S., Sorokin, D.Y., Verbarg, S., Rohde, M., Woyke, T. and Kyrpides, N.C., 2019. Sulfate-reducing bacteria that produce exopolymers thrive in the calcifying zone of a hypersaline cyanobacterial mat. *Frontiers in Microbiology*, *10*, 862.

Staley, J.T., Irgens, R.L. and Brenner, D.J., 1987. *Enhydrobacter* *aerosaccus* gen. nov., sp. nov., a gas-vacuolated, facultatively anaerobic, heterotrophic rod. *International Journal of Systematic and Evolutionary Microbiology*, *37*, 289-291.

Stetter, K.O., König, H. and Stackebrandt, E., 1983. *Pyrodictium* gen. nov., a new genus of submarine disc-shaped sulphur reducing archaebacteria growing optimally at 105°C. *Systematic and Applied Microbiology*, *4*, 535-551.

Stieglmeier, M., Klingl, A., Alves, R.J., Simon, K.M.R., Melcher, M., Leisch, N. and Schleper, C., 2014. *Nitrososphaera* *viennensis* gen. nov., sp. nov., an aerobic and mesophilic, ammonia-oxidizing archaeon from soil and a member of the archaeal phylum Thaumarchaeota. *International Journal of Systematic and Evolutionary Microbiology*, *64*, 2738.

Stohr, R., Waberski, A., Völker, H., Tindall, B.J. and Thomm, M., 2001. *Hydrogenothermus* *marinus* gen. nov., sp. nov., a novel thermophilic hydrogen-oxidizing bacterium, recognition of *Calderobacterium* *hydrogenophilum* as a member of the genus *Hydrogenobacter* and proposal of the reclassification of *Hydrogenobacter* *acidophilus* as *Hydrogenobaculum* *acidophilum* gen. nov., comb. nov., in the phylum'Hydrogenobacter/Aquifex'. *International Journal of Systematic and Evolutionary Microbiology*, *51*, 1853-1862.

Su, C.C., Deng, W.L., Jan, F.J., Chang, C.J., Huang, H., Shih, H.T. and Chen, J., 2016. *Xylella* *taiwanensis* sp. nov., causing pear leaf scorch disease. *International Journal of Systematic and Evolutionary Microbiology*, *66*, 4766-4771.

Subhash, Y., Sasikala, C. and Ramana, C.V., 2014. *Pontibacter* *ruber* sp. nov. and *Pontibacter* *deserti* sp. nov., isolated from the desert. *International Journal of Systematic and Evolutionary Microbiology*, *64*, 1006-1011.

Sun, X., Luo, P. and Li, M., 2015. *Paracoccus* *angustae* sp. nov., isolated from soil. *International Journal of Systematic and Evolutionary Microbiology*, *65*, 3469-3475.

Sun, X.W., Abdugheni, R., Huang, H.J., Wang, Y.J., Jiang, M.Z., Liu, C., Zhou, N., Jiang, H. and Liu, S.J., 2022. *Bacteroides* *propionicigenes* sp. nov., isolated from human faeces. *International Journal of Systematic and Evolutionary Microbiology*, *72*, 005397.

Suresh, K., Mayilraj, S., Bhattacharya, A. and Chakrabarti, T., 2007. *Planococcus* *columbae* sp. nov., isolated from pigeon faeces. *International Journal of Systematic and Evolutionary Microbiology*, *57*, 1266-1271.

Takai, K., Komatsu, T. and Horikoshi, K., 2001. *Hydrogenobacter* *subterraneus* sp. nov., an extremely thermophilic, heterotrophic bacterium unable to grow on hydrogen gas, from deep subsurface geothermal water. *International Journal of Systematic and Evolutionary Microbiology*, *51*, 1425-1435.

Vancanneyt, M., Segers, P., Torck, U., Hoste, B., Bernardet, J.F., Vandamme, P. and Kersters, K., 1996. Reclassification of *Flavobacterium* *odoratum* (Stutzer 1929) Strains to a New Genus, *Myroides*, as *Myroides* *odoratus* comb. nov. and *Myroides* *odoratimimus* sp. nov. *International Journal of Systematic and Evolutionary Microbiology*, *46*, 926-932.

Vandamme, P., De Brandt, E., Houf, K. and De Baere, T., 2012. *Kerstersia* *similis* sp. nov., isolated from human clinical samples. *International Journal of Systematic and Evolutionary Microbiology*, *62*, 2156-2159.

Vandamme, P.A., Peeters, C., Inganäs, E., Cnockaert, M., Houf, K., Spilker, T., Moore, E.R. and LiPuma, J.J., 2016. Taxonomic dissection of *Achromobacter* *denitrificans* Coenye et al. 2003 and proposal of *Achromobacter* *agilis* sp. nov., nom. rev., *Achromobacter* *pestifer* sp. nov., nom. rev., *Achromobacter* *kerstersii* sp. nov. and *Achromobacter* *deleyi* sp. nov. *International Journal of Systematic and Evolutionary Microbiology*, *66*, 3708-3717.

Vela, A.I., Collins, M.D., Lawson, P.A., García, N., Domínguez, L. and Fernández-Garayzábal, J.F., 2005. *Uruburuella* *suis* gen. nov., sp. nov., isolated from clinical specimens of pigs. *International Journal of Systematic and Evolutionary Microbiology*, *55*, 643-647.

Vogel, B.F., Venkateswaran, K., Christensen, H., Falsen, E., Christiansen, G. and Gram, L., 2000. Polyphasic taxonomic approach in the description of *Alishewanella* *fetalis* gen. nov., sp. nov., isolated from a human foetus. *International Journal of Systematic and Evolutionary Microbiology*, *50*, 1133-1142.

Wang, C.Y., Ng, C.C., Tzeng, W.S. and Shyu, Y.T., 2009. *Marinobacter* *szutsaonensis* sp. nov., isolated from a solar saltern. *International Journal of Systematic and Evolutionary Microbiology*, *59*, 2605-2609.

Wang, Y., Song, J., Zhai, Y., Zhang, C., Gerritsen, J., Wang, H., Chen, X., Li, Y., Zhao, B., Zhao, B. and Ruan, Z., 2015. *Romboutsia* *sedimentorum* sp. nov., isolated from an alkaline-saline lake sediment and emended description of the genus *Romboutsia*. *International Journal of Systematic and Evolutionary Microbiology*, *65*, 1193-1198.

Wang, Z., Cheng, J.H., Zhang, X.Y., Xu, F., Fu, H.H., Li, C.Y., Zhang, Y.Z., Chen, X.L., Song, X.Y. and Zhang, X.Y., 2022. *Arsukibacterium* *indicum* sp. nov., isolated from deep-sea sediment, and transfer of *Rheinheimera* *tuosuensis* and *Rheinheimera* *perlucida* to the genus *Arsukibacterium* as *Arsukibacterium* *tuosuense* comb. nov. and *Arsukibacterium* *perlucidum* comb. nov. *International Journal of Systematic and Evolutionary Microbiology*, *72*, 005455.

Williams, R.E.O., Hirch, A. and Cowan, S.T., 1953. *Aerococcus*, a new bacterial genus. *Microbiology*, *8*, 475-480.

Xi, L., Qiao, N., Liu, D., Li, J., Zhang, J. and Liu, J., 2018. *Pannonibacter* *carbonis* sp. nov., isolated from coal mine water. *International Journal of Systematic and Evolutionary Microbiology*, *68*, 2042-2047.

Xu, L., Shi, J.F., Zhao, P., Chen, W.M., Qin, W., Tang, M. and Wei, G.H., 2011. *Rhizobium* *sphaerophysae* sp. nov., a novel species isolated from root nodules of *Sphaerophysa* *salsula* in China. *Antonie Van Leeuwenhoek*, *99*, 845-854.

Yabuuchi, E., Kaneko, T., Yano, I., Moss, C.W. and Miyoshi, N., 1983. *Sphingobacterium* gen. nov., *Sphingobacterium* *spiritivorum* comb. nov., *Sphingobacterium* *multivorum* comb. nov., *Sphingobacterium* *mizutae* sp. nov., and *Flavobacterium* *indologenes* sp. nov.: glucose-nonfermenting gram-negative rods in CDC groups IIK-2 and IIb. *International Journal of Systematic and Evolutionary Microbiology* 33, 580-598.

Yabuuchi, E., Kosako, Y., Yano, I., Hotta, H. and Nishiuchi, Y., 1995. Transfer of two *Burkholderia* and an *Alcaligenes* species to *Ralstonia* gen. nov.: proposal of *Ralstonia* *pickettii* (Ralston, Palleroni and Doudoroff 1973) comb. nov., *Ralstonia* *solanacearum* (Smith 1896) comb. nov. and *Ralstonia* *eutropha* (Davis 1969) comb. nov. *Microbiology and Immunology*, *39*, 897-904.

Yang, C., Bai, Y., Dong, K., Yang, J., Lai, X.H., Lu, S., Zhang, G., Cheng, Y., Jin, D., Zhang, S. and Lv, X., 2021. *Actinomyces* *marmotae* sp. nov. and *Actinomyces* *procaprae* sp. nov. isolated from wild animals and reclassification of *Actinomyces* *liubingyangii* and *Actinomyces* *tangfeifanii* as *Boudabousia* *liubingyangii* comb. nov. and *Boudabousia* *tangfeifanii* comb. nov., respectively. *International Journal of Systematic and Evolutionary Microbiology*, *71*, 004696.

Yang, F., Liu, H.M., Zhang, R., Chen, D.B., Wang, X., Li, S.P. and Hong, Q., 2015. *Chryseobacterium* *shandongense* sp. nov., isolated from soil. *International Journal of Systematic and Evolutionary Microbiology*, *65*, 1860-1865.

Yang, G., Chen, M., Yu, Z., Lu, Q. and Zhou, S., 2013a. *Bacillus* *composti* sp. nov. and *Bacillus* *thermophilus* sp. nov., two thermophilic, Fe (III)-reducing bacteria isolated from compost. *International Journal of Systematic and Evolutionary Microbiology*, *63*, 3030-3036.

Yang, G., Han, L., Wen, J. and Zhou, S., 2013b. *Pseudomonas* *guangdongensis* sp. nov., isolated from an electroactive biofilm, and emended description of the genus *Pseudomonas* Migula 1894. *International Journal of Systematic and Evolutionary Microbiology*, *63*, 4599-4605.

Yang, L., Muhadesi, J.B., Wang, M.M., Wang, B.J., Liu, S.J. and Jiang, C.Y., 2018. *Thauera* *hydrothermalis* sp. nov., a thermophilic bacterium isolated from hot spring. *International Journal of Systematic and Evolutionary Microbiology*, *68*, 3163-3168.

Yoshizawa, S., Karatani, H., Wada, M., Yokota, A. and Kogure, K., 2010. *Aliivibrio* *sifiae* sp. nov., luminous marine bacteria isolated from seawater. *The Journal of General and Applied Microbiology*, *56*, 509-518.

You, J., Li, Y., Hong, S., Wang, J., Yu, J., Mu, B., Ma, X. and Xue, Y., 2019. *Tepidicella* *baoligensis* sp. nov., a novel member of betaproteobacterium isolated from an oil reservoir. *Current Microbiology*, *76*, 410-414.

Yu, Z., Zhuang, L., Pan, J., Wang, Y. and Zhou, S., 2016. *Nitratireductor* *lacus* sp. nov., isolated from Yuncheng Salt Lake, China. *International Journal of Systematic and Evolutionary Microbiology*, *66*, 4963-4967.

Zavarzina, D.G., Zhilina, T.N., Tourova, T.P., Kuznetsov, B.B., Kostrikina, N.A. and Bonch-Osmolovskaya, E.A., 2000. *Thermanaerovibrio* *velox* sp. nov., a new anaerobic, thermophilic, organotrophic bacterium that reduces elemental sulfur, and emended description of the genus *Thermanaerovibrio*. *International Journal of Systematic and Evolutionary Microbiology*, *50*, 1287-1295.

Zhang, H., Wang, H., Chen, H., Sun, Q., Zhong, Z., Wang, M., Cao, L., Lian, C., Zhou, L. and Li, C., 2020. *Nitrincola* *iocasae* sp. nov., a bacterium isolated from sediment collected at a cold seep field in the South China Sea. *International Journal of Systematic and Evolutionary Microbiology*, *70*, 4897-4902.

Zhang, H., Zhang, J., Song, M., Cheng, M.G., Wu, Y.D., Guo, S.H., Li, Q., Hong, Q. and Huang, X., 2015. *Pedobacter* *nanyangensis* sp. nov., isolated from herbicide-contaminated soil. *International Journal of Systematic and Evolutionary Microbiology*, *65*, 3517-3521.

Zhang, J., Wang, J., Fang, C., Song, F., Xin, Y., Qu, L. and Ding, K., 2010. *Bacillus* *oceanisediminis* sp. nov., isolated from marine sediment. *International Journal of Systematic and Evolutionary Microbiology*, *60*, 2924-2929.

Zhou, Y., Gao, X., Xu, J., Li, G., Ma, R., Yan, P., Dong, C. and Shao, Z., 2021. *Mesonia* *hitae* sp. nov., isolated from the seawater of the South Atlantic Ocean. *International Journal of Systematic and Evolutionary Microbiology*, *71*, 004911.

**Supplementary Figure**

| 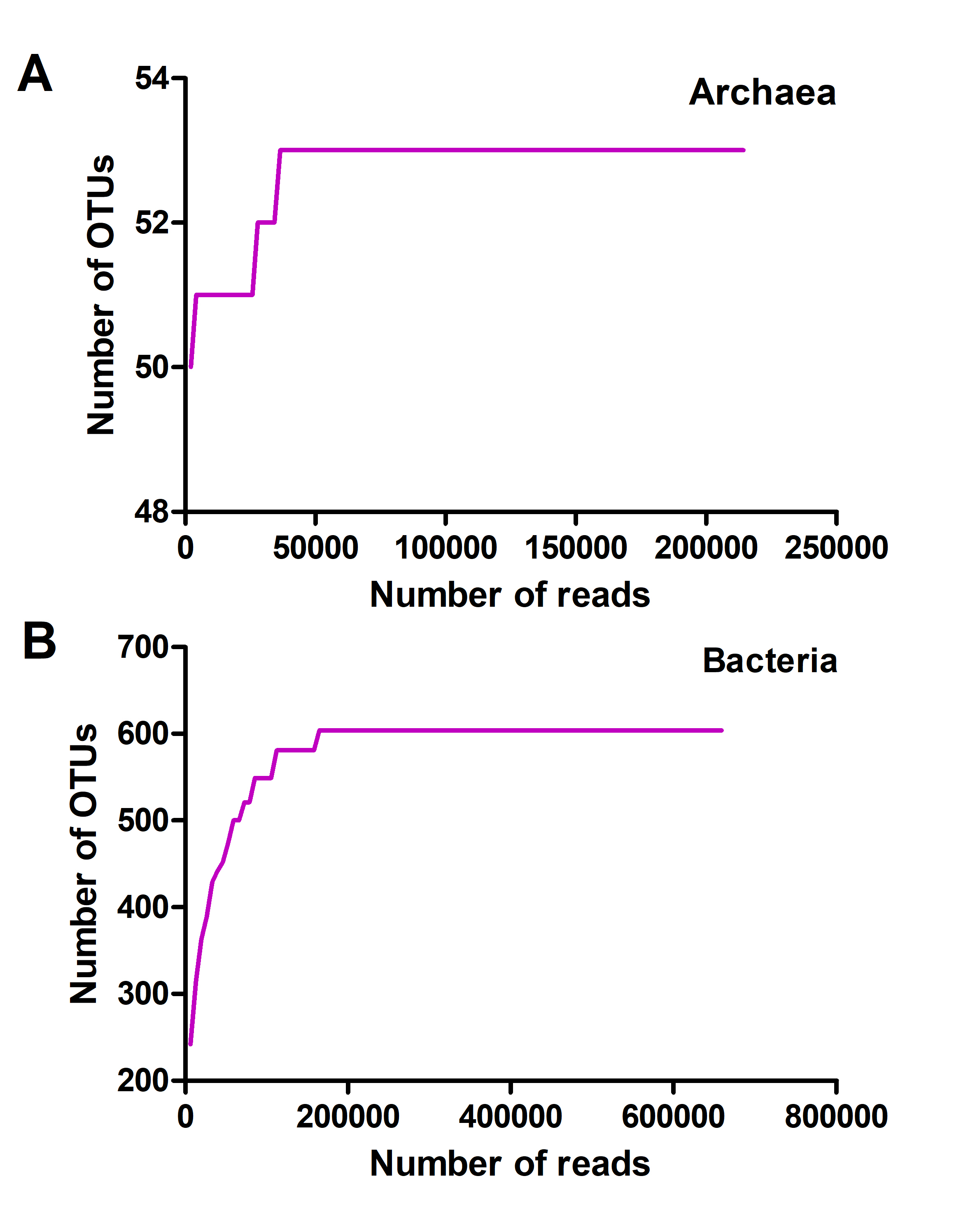 |
| --- |
| **Fig S1.** Rarefaction curves showing the proportionality between OTU-level diversity revealed and the number of 16S rRNA gene sequence reads analyzed, for (**A**) archaea or (**B**) bacteria, in the vent-water sample. For each sample, the number of reads used in OTU-clustering is plotted along the X-axis, while number of OTUs created is plotted along the Y-axis. |
