# Supplementary material for: Aquificae overcomes competition by archaeal thermophiles, and crowding by bacterial mesophiles, to dominate the boiling vent-water of a Trans-Himalayan sulfur-borax spring
Source: PLoS One. 2024 Oct 25;19(10):e0310595. doi: 10.1371/journal.pone.0310595 (PMC11508158; doi:10.1371/journal.pone.0310595)
Supplement: Attachment — Submitted filename: The paper by Mondal et al.docx [file pone.0310595.s003.docx]

The paper by Mondal et al. is devoted to the microbiome of the hot spring (namely, geyser), called Lotus Pond, located in the Puga Valley on the Changthang plateau. The authors applied the metagenomics analysis, including MAGs binning and their functional analysis, 16S rRNA amplicon sequencing and enumeration of microbial cells, filtered out from the hot spring water, with different fluorescent dyes. The main hypotheses of this work are:

- that genomes of *Aquificota* possess many genes of antibiotic biosynthesis and even virulence functions, as well as antibiotic resistance genes, that helps them dominating in the hot spring, overlapping the other thermophiles, especially Archaea;
- and bacterial mesophiles are not random components of this hot spring community and their genomes are replete with genes crucial for hydrothermal and other stress adaptation;

However, the data, obtained by *in silico* analysis of MAGs, does not support these conclusions. Specifically:

- In the article the authors did not indicate the method by which the functional analysis of the metagenome was performed. As I realized from the manuscript, the metagenome was annotated only as CDSs by searching against the eggNOG database. But this is the first step in *in silico* analysis and it can only reveal general, "raw" things about metabolism of community’s components. The functions revealed in this way only provide directions in which researchers can ‘dig’ further and deeper to understand the nature of what is going on. Relying only on such analysis is very naive and rather superficial.
- Secondary metabolites biosynthesis clusters are usually identified by antiSMASH program (doi:10.1038/nrmicro3330) with strict search parameters. Homologues must be found for each identified possibility. We need to realize that we are only talking about secondary metabolites (these are not equal to “antibiotic compounds”), and they can perform many different functions in prokaryote metabolism. In addition, it is necessary for proofing to find genes for all proteins of antibiotic compound biosynthesis, only fragments are not enough. It is also desirable to identify genes encoding transporters, regulating enzymes and so on. In ideal case it is necessary to confirm the assumptions by ‘wet’ experiments or at least references to the literature. And I am not aware of any cases (*in silico* or *in vitro*) of identification of antimicrobial activity in *Aquificota* representatives. The same situation with genes with antibiotic resistance. Usually the ABRicate tool (https://github.com/tseemann/abricate) or ResFinder (doi: 10.1093/jac/dkaa345) and the CARD database (doi:10.1128/AAC.00419-13) are used for such analysis. The analysis in Mondal et al. is based on CDSs predictions only, which is too general and not precise.
- The authors postulated that the *Aquificota* acted as the key primary producers of the ecosystem of Lotus Pond, since genes of rTCA cycle were found in MAGS affiliated to *Hydrogenobacter* close relative. First, the presence of rTCA in *Aquificota* and their role as primary producers in hot spring communities is a long-known fact; second, the genes found in MAGs in this work cannot strictly relate to the rTCA, since they all relate to the TCA or even to ‘exit’ proteins from this pathway. To distinguish TCA from rTCA, one reaction must be found: instead of citrate synthase, a gene for citryle-CoA lyase/ATP-citrate lyase must be present. This is explained in details in the Berg et al., 2011 article referenced by the authors. And a small addition, not relating to the serious inaccuracies of the work, but showing not a full knowledge of the issues discussed there, that nowadays 7 (or even 9, depending on the scientists' opinion) pathways of autotrophic CO_2_ assimilation are already known (DOI: [10.1126/science.aao2410](https://doi.org/10.1126/science.aao2410); DOI: [10.1126/science.aao3407](https://doi.org/10.1126/science.aao3407); doi/10.1073/pnas.1904225116; doi: 10.1038/s41467-020-18906-7), and new pathways have also been identified in the representatives of *Aquificota*.
- The work focuses very much on the diversity, relative abundance and capabilities of mesophilic prokaryotes in analyzed extremely hot spring (85°C). However, estimating their representation in the community by CDSs gave an inadequate result, because: 1) it doesn't mention what method was used to determine that; 2) the genome sizes of thermophilic prokaryotes belonging to such truly deep-phylogenetic lineages, such as *Aquificota*, *Thermotogota*, *Thermoproteota* etc., are 2-3 times smaller than those of mesophilic bacteria. Accordingly, the number of genes in genomes of thermophiles is much less. Herewith the 16S gene metagenomic analysis reflected the usual pattern for such conditions quite properly, where *Aquificota* vastly dominated over the others. Moreover, the stained cells counting experiment (by the way, it was completely absent in the Discussion) revealed approximately 63,5% (5,4/8,5*100%) of metabolically viable cells, that could be all truly thermophiles. No evidence was given to prove that mesophiles could contribute to living cells quantity. Real time PCR, or FISH with specific probes engineering to mesophilic taxons, or cultivation of water/filter sample in mesophilic conditions and enriching any mesophilic culture (with strict controls, of course) might help in this situation. And the additional research on 16S amplicons with different primers only confuses the reader. It is not very clear to me why it was necessary to carry out such an analysis if it has already been done in previous works by the same group of authors. This article is not a methodological work, so why authors devoted so much time and efforts to comparison them?
- Presence of mesophilic DNA in the sample from the hot spring with 85°C can be explained by the following factors: 1) Dirty sampling; 2) Low concentration or low quality of the total DNA (the authors did not provide any evidence to the contrary, and the low cell counts in the study – 10^4^ cells/ml - may indicate at least a low amount of DNA in the analysis); 3) Mesophiles may be transferred accidentally to hot spring from cold habitats nearby with precipitation, plant fall, river, etc. In the case of Lotus Pond, a geyser, each time discharging to the surface, may capture nearby cold areas and flush mesophiles into itself with a strong stream of water. But in any case mesophiles cannot be considered as true components of the hot spring community. This is supported by the facts that there are no pure cultures of mesophiles capable of growing (not surviving for a short period of time) at 85°C, and I don’t know any ecological articles devoted to microbial communities of other hydrothermal systems with such extreme temperatures (like in YNP, Iceland, Russia, Japan hot springs) where it was proven that mesophiles were there not by a chance. In this research, the authors provided references only by their group’s works and only in this region. But this is a little confusing.

I think it's an interesting idea that *Aquificota* may contain in their genomes a number of genes for the synthesis of antibiotic compounds, which could inhibit the growth of other thermophiles and help them to dominate in almost all hydrothermal areas worldwide. And if the authors decide to prove this hypothesis, this study requires additional experiments, particularly proper *in silico* analysis of MAGs and/or genomes and *in vitro* antibiotic activities of pure cultures of *Aquificota* at least. And in this case it will be a truly novel bright work. But in the meantime the work is too surface level, despite the time and efforts put into writing it. The results of the analysis presented in the article do not support the title or what is put in the abstract.
