# Supplementary material for: Aquificae overcomes competition by archaeal thermophiles, and crowding by bacterial mesophiles, to dominate the boiling vent-water of a Trans-Himalayan sulfur-borax spring
Source: PLoS One. 2024 Oct 25;19(10):e0310595. doi: 10.1371/journal.pone.0310595 (PMC11508158; doi:10.1371/journal.pone.0310595)
Supplement: Attachment — Submitted filename: Response to Reviewers.doc [file pone.0310595.s004.doc]

**Response to the comments of Reviewer 1**

I wish to express my gratitude to the author for their diligent efforts in conducting the study titled "Antibiosis helps Aquificae surmount competition by archaeal thermophiles, and crowding by bacterial mesophiles, to dominate the boiling vent-water of a Trans-Himalayan sulfur-borax spring". It contributes to enhance our understanding on functional profile of microbiome from geothermal region. However, there is a significant need for improvement in the method section, particularly considering the nature of the sequencing platform. All claims in the study rely solely on results from gene calling, assembly, taxonomic assignment, and functional annotation. Substantial enhancements in the methodological approach would strengthen the study's validity and reliability.

RESPONSE: We thank the Reviewer for appreciating our detailed exploration of the microbiome architecture of the geobiologically unique Trans-Himalayan sulfur-borax spring system.

We agree with the suggestions pertaining to the strengthening of the methods section, so have now dealt with these concerns primarily by bringing to the main text all those procedural details that were previously narrated in the Supplementary Methods. Thus, in the revised Article File,

we have now elaborated the previous Methods section called “Metagenomics” under three new sections titled as

- Metagenome extraction and sequencing
- Assembly and annotation of the metagenomic sequence
- Direct annotation of metagenomic reads corresponding to rRNA gene sequences

besides significantly substantiating the Methods sections

- Construction and taxonomic characterization of metagenome-assembled genomes (MAGs)
- PCR-amplified 16S rRNA gene sequence analysis.

I have outlined certain aspects that require attention from the authors.

I encountered difficulty in understanding the English used, particularly in the abstract and introduction. Sentences are excessively long, featuring unnecessary words and a lack of coherence between phrases separated by commas. Please aim for clarity and conciseness.

RESPONSE: We agree with the Reviewer’s concerns regarding the apparently difficult English used in certain sections of the manuscript. We have now tried our best to make the sentences simple and short, yet comprehending some of them may still require additional attention. The geochemical distinctiveness of the hot spring explored and the uniqueness of the microbiome thriving therein are both quite complex. Accordingly, the intricate issues pertaining to the geomicrobiological peculiarities of Trans-Himalayan sulfur-borax springs, compared with other well studied hydrothermal ecosystems, need to be put in their proper perspectives without over-simplification.

The author utilized the eggNOG database for functional analysis, last updated in 2019. However, it's worth noting that the COG database (<https://www.ncbi.nlm.nih.gov/research/cog>) is the most recent, updated in March 2022. The COG database provides dedicated resources for both bacteria and archaea. I strongly recommend that the author considers using the COG database for more up-to-date and comprehensive functional profiling.

RESPONSE: We agree, so have now removed all COG-related information from the eggNOG-derived annotation of CDSs shown in Table S2 (previously numbered as Table S1), and provided fresh data obtained by searching the Prodigal-derived putatively translated gene catalog against the COG Little Endian version of the Conserved Domain Database of NCBI located at <https://ftp.ncbi.nih.gov/pub/mmdb/cdd/little_endian/Cog_LE.tar.gz>, using COGclassifier v1.0.5 (please see lines 241-245, 498-517 of the Track Changes file).

The author might want to consider including Quality Check (QC) results of the FASTQ files in the supplementary section, both before and after read filtration. This additional information can offer insights into the data quality and the impact of the filtration process.

RESPONSE: We agree, so have now included Quality Check (QC) results for the raw read set as well as the one obtained after quality filtering (please see the new Table S1 of the Revised Manuscript).

Consistency should maintain in providing parameter information for the tools used in the analysis. For example parameter information for Prodigal v2.6.3 is missing. If default parameters were used, please mention this in the documentation.

RESPONSE: We agree and have now included the parameter information for all the bioinformatic software programs that have been used in this study, including Prodigal v2.6.3 (please see lines 197-321 of the Track Changes file).

The study references the searching of reads against the rrnDB database, a crucial step for taxonomic delineation. However, there is limited information on read mapping and extraction. It would be beneficial if the author could provide a detailed workflow, including steps, parameters, and intermediate files used for the mapping and extraction of reads. Additionally, if any other tools were employed in this process, their inclusion as an workflow would be appreciated.

RESPONSE: We agree and have now added the warranted details of read mapping, extraction, and classification, to the new Methods section titled “Direct annotation of metagenomic reads corresponding to rRNA gene sequences” (please see lines 254-261 of the Track Changes file).

Given that the Shotgun sequence is not the most suitable option for taxonomic profiling with publicly available 16S databases, I suggest that the authors utilize a dedicated pipeline for shotgun metagenome data, such as MEGAN Community Edition.

RESPONSE: We used the MEGAN6 Community Edition to taxonomically classify the metagenomic reads that were detected by searching the raw dataset against the SILVA_SSURef_138.1_NR99 database with the help of Minimap2-2.26 (Li, 2018). Curiously, this led to the detection of no read ascribed to Crenarchaeota / Thermoproteota, Thermotogae / Thermotogota, Cyanobacteria, Chloroflexi / Chloroflexota, Actinobacteria / Actinomycetota, Synergistetes / Synergistota, even though 396, 196, 131, 56, 21, and 2 reads were detected for these taxa respectively, in our existing RDP-based analyses. Betaproteobacteria and Alphaproteobacteria, for which 1345 and 595 reads were identified in the RDP-based annotation, also went undetected in the MEGAN-based analysis. In fact, no phylum other than Aquificota, Proteobacteria (only the Gamma class), Firmicutes, Deinococcota, Bacteroidota were detected in the MEGAN6-based classification.

Given that the metagenome under consideration is that of a boiling vent-water community, *in situ* presence of phyla such as Thermoproteota (this included genera such as *Aeropyrum*, *Pyrobaculum* and *Stetteria*) and Thermotogota, as detected in the existing RDP-based classification, is obviously true; accordingly, a bioinformatic tool which fails to identify these native taxa cannot constitute a technique of choice in the present case.

In the revised manuscript, therefore, we retain the contextually more acceptable RDP-based classification of raw metagenomic reads corresponding to 16S rRNA genes.

The study employed the UBCG Pipeline v3.0 for core gene phylogeny, which has limitations in identifying less core gene due to its reliance on a universal core genome database. Consequently, it overlooked several actual core genes in the user dataset. I recommend utilizing the best-bidirectional hits (BBH) method for core gene identification, as it is sure to offer a more comprehensive core gene set from the given genomic dataset.

RESPONSE: We agree that the UBCG Pipeline identifies relatively less number of core genes due to its reliance on the universal core gene database. However, since the MAG dataset involved in the current analysis includes entities diverged at the level of the phylum, we have no other choice but to use the universal core gene database (hence UBCG) to accommodate all the MAGs in one phylogenetic tree. Furthermore, when we used Roary - The pan genome pipeline v3.23.0, as well as GET_HOMOLOGUES v3.6.2, to identify core genes using the best-bidirectional hits (BBH) method, we were unable to identify any gene with a conservation level of >90% sequence similarity across the MAGs.

MAGs with accession numbers JARJND000000000 and JARJNC000000000 exhibit a completeness of less than 50% in Table-1, indicating significant incompleteness and poor quality. It is advisable to exclude these MAGs from the analysis for more robust and reliable results.

RESPONSE: It’s true that the completeness levels of the MAGs having accession numbers JARJND000000000 and JARJNC000000000 are 30% and 34% respectively. However, it is noteworthy that the two MAGs have contamination levels of only 0.3% and 1.1% respectively, so they cannot be regarded as being poor in quality.

In the current analysis, 19, 14, and 30 MAGs were initially generated using Metabat2, MaxBin2, and CONCOCT, respectively. Comprehensive refinement and optimization of the 63 population genome bins taken together, using DASTool, finally qualified 15 MAGs as the best representatives of the metagenome, notwithstanding the low completeness level of a few shortlisted MAG. This result needs to be presented upfront in its entirety without any modification/subtraction of convenience. This is important for the sake of the comprehensiveness as well as the integrity of the data.

Moreover, in the context of the microbiome, it is most important to note that the two MAGs JARJND000000000 and JARJNC000000000 belonged to the family Thermodesulfobacteriaceae and the genus *Paracoccus* respectively. Thermodesulfobacteria is the most predominant taxon of true thermophiles within the Lotus Pond ecosystem after Aquificae, while *Paracoccus* strains isolated during previous expeditions of Lotus Pond have been studied in-depth for their thermal endurance (Mondal et al. 2022: *Microbiology Spectrum* 10, e01606-22).

All the above issues strongly support the essentiality of a robust documentation of the two MAGs in the present manuscript, even if their completeness is currently low (notably, generation of more metagenomic sequence data from this sample in the future can well increase the completeness of the two MAGs).

**Response to the comments of Reviewer 2**

This manuscript presents data from a unique ecosystem that is understudied and worthy of better characterisation. For that reason, I am recommending a major revision before this manuscript can be published.

I had a hard time reading this manuscript due both to the way it is written and the choice of data analysis and visualisation.

RESPONSE: We agree, and believe that the Reviewer appreciated our detailed exploration, as well as the underlying phenomenology, of the microbiome architecture of the geobiologically unique Trans-Himalayan hot spring system. As suggested, we have now overhauled the manuscript by focusing on the main findings and making the overall text (especially the language) more lucid.

The manuscript is excessively long, which compromises readability. Throughout the paper the tendency for long sentences, that usually focus on different ideas, is maintained. In many sentences the choice of words is unusual, to say the least.
As an example of what I am saying the sentence in the Introduction:
"Idiosyncratic to the above community structure axiomatic for hydrothermal ecosystems" - what is an axiomatic ecosystem, and what idiosyncrasy are you referring to?

RESPONSE: We have now tried our best to abridge the text, remove the asides, and make the sentences simple and short. Yet following some of the sentences may still require additional attention. The geochemical distinctiveness of the hot spring explored and the uniqueness of the microbiome thriving therein are both quite complex. Accordingly, the issues pertaining to their peculiarities (idiosyncrasies), compared with other well studied hydrothermal systems and their microbiota, need to be put in their proper perspectives without over-simplification.

As for the sentence in question, we have now assorted the information into two separate sentences, and added precise terminologies (such as “low-biodiversity community structure” instead of “above community structure”, and “extra-ordinarily diversified vent-water microflora” instead of “extra-ordinarily high habitability”) to bring more clarity to the message conveyed (please see lines 93-98 of the Track Changes file).

In relation to the Reviewer’s queries “what is an axiomatic ecosystem", and “what idiosyncrasy had been referred to”, a careful reading of the previous sentence

“Idiosyncratic **to the above** community structure axiomatic **for** hydrothermal ecosystems, a geochemically unusual category of Trans-Himalayan hot springs …... have been reported to feature extra-ordinarily high habitability”

points out that

- no ecosystem is being called axiomatic here but the typical community structure described in the previous paragraph is being termed as **axiomatic for hydrothermal ecosystems** in general (please note the preposition “**for**” after the word “axiomatic”);
- and, the extra-ordinarily high habitability of Trans-Himalayan hot springs is being referred to as idiosyncratic **to the aforesaid** community structure that in turn is axiomatic for hydrothermal ecosystems (in this case the preposition “to” after the word “idiosyncratic” is to be noted).

Please consider getting someone to help revising the English. As it is, it reads as if a machine has tried to come up with dictionary synonyms for words that are more commonly used, even in scientific writing.

RESPONSE: We have now considerably shortened the text by deleting the issues of tangential importance (please see lines 65-80, 646-653, 686-692, 746-798, 838-876, 886-913, 1182-1237 of the Track Changes file). The new text focuses on the main findings and their implications. We have also made the sentences simpler by splitting them wherever possible. That said, some sentences may still found complicated and warrant additional attention as the issues dealt there are complex and cannot be oversimplified.

There is a lack of detail in the Material and Methods section regarding data analysis and visualisation. You detail how you get MAGs and OTUs and then no more information is provided. After you get the MAGs and the 16S reads how do you analyse them? For example, compare the legend in Fig 3 and Fig 6, the latter explains the analysis that was done.

RESPONSE: In the previous manuscript, all the details of the bioinformatic procedure were given in the Supplementary Methods. We have now brought all those critical information to the main text to add to the reader’s satisfaction as well as the study's reliability (please see lines 167-321 of the Track Changes file).

Thus, in the revised Article File,

we have now elaborated the previous Methods section called “Metagenomics” under three new sections titled as

- Metagenome extraction and sequencing
- Assembly and annotation of the metagenomic sequence
- Direct annotation of metagenomic reads corresponding to rRNA gene sequences,

besides significantly substantiating the Methods sections

- Construction and taxonomic characterization of metagenome-assembled genomes (MAGs)
- PCR-amplified 16S rRNA gene sequence analysis (for the upgraded methods pertaining to MAGs and OTUs please see lines 263-321 of the Track Changes file).

Also asking as this is not the most common way of presenting these data. It is also very uninformative, as none of your Figures help to visualise the conclusions you draw from your data. Figure 5 is particularly unhelpful, and if I understood correctly the only thing that is related to your work is the legend?

RESPONSE: In the revised manuscript,

Figure 1 depicts the topography of the study site. Figure 2 shows representative picture of the fluorescence microscopic fields based on which microbial cell density was calculated in the vent-water sample of Lotus Pond. Figure 3 illustrate the overall microbiome composition by providing an overview of the taxonomic distribution of (A) the CDSs identified in the assembled metagenome of Lotus Pond, and (B) the metagenomic reads which corresponded to 16S rRNA genes. Figure 4 presents the phylogenetic tree that delineated the evolutionary relationships among the 14 bacterial MAGs reassembled from the Lotus Pond vent-water metagenome, and their closest genomic relatives. Figure 5 gives a simplified schematic view of the carbon assimilation pathways for which genes were identified in the assembled metagenome of Lotus Pond. Table 1 enumerates the key features of the MAGs, while Table 2 lists those bacterial genera which were detected in the present exploration of the Lotus Pond vent-water as well as in one or more previous studies of the same habitat. We believe all these 7 documents help the paper achieve its professed objective of getting a quantitative idea about the microbiome architecture.

Figure 5 is specifically essential to document which genes were identified, and in what numbers, in the assembled metagenome of Lotus Pond for each major carbon assimilation pathway. We believe this schematic diagram incorporate in the simplest and shortest possible way all the relevant qualitative and quantitative data that were mined from the metagenome in relation to carbon fixation pathways.

Your data analysis and visualisation should show and support the main points you make in your conclusions. As an example, in your title you use the word Antibiosis, but nowhere in the paper do you show that is possible. The fact that you find a potential gene does not mean that it is being expressed. You have no data from RNA, therefore any activity must be considered as potential. Either you do that, or you rephrase the manuscript to reflect the data analysis that you are presenting.

RESPONSE: We agree, so have now significantly downsized the metagenomic data mining results and corresponding discussions on antibiosis (This included removal of Figure 6). We have also removed the issue altogether from the title, and explicitly mentioned in the few remaining lines of relevant discussion that any activity of the Lotus Pond microflora in this regard is purely potential and subject to actual experimental verification via future studies of culture-dependent microbiology and gene expression analysis (please see lines 59-80, 816-913, 1182-1237, 1270-1275 of the Track Changes file).

In relation to the conspicuous absence of metatranscriptomic data, which are essential to ascertain all the community metabolism predicted in this study, it is noteworthy that the elucidation of the microbiome structure and functions for the boiling vent-waters of Trans-Himalayan sulfur-borax hot springs is an ongoing process. We had previously outlined the alpha diversity of the system via analysis of amplified 16S rRNA gene sequences, here we explored the microbiome via deep shotgun metagenomics, and in the very near future we shall communicate the detailed metatranscriptome analyses for this unique microbiome.

Please note that these comments are meant to be read as examples of issues with the whole manuscript, which should be thoroughly revised to improve readability, reduce length and provide data visualisation and analysis that is in line with the most common methods.

RESPONSE: We agree, so have now comprehensively revised the manuscript by focusing on improving the readability, and reducing the length, of the text (please see lines 65-80, 646-653, 686-692, 746-798, 838-876, 886-913, 1182-1237 of the Track Changes file). However, so far as providing data visualization and analyses based on common methods is concerned it needs to be appreciated that the phenomenology underlying this microbiome exploration is not commonplace. The geochemical distinctiveness of the hot spring explored, and the uniqueness of the microbiome thriving therein, are both quite complex. Therefore, putting the geomicrobiological peculiarities of the Trans-Himalayan sulfur-borax springs, in comparison with other well-studied hydrothermal ecosystems, entails a lot of intricate textual deliberations. Such a complex system cannot be documented by routine software-generated display items that we habitually find in most metagenomics-based papers of microbiome exploration.

**Response to the comments of Reviewer 3**

The paper by Mondal et al. is devoted to the microbiome of the hot spring (namely, geyser), called Lotus Pond, located in the Puga Valley on the Changthang plateau. The authors applied the metagenomics analysis, including MAGs binning and their functional analysis, 16S rRNA amplicon sequencing and enumeration of microbial cells, filtered out from the hot spring water, with different fluorescent dyes. The main hypotheses of this work are:

- that genomes of *Aquificota* possess many genes of antibiotic biosynthesis and even virulence functions, as well as antibiotic resistance genes, that helps them dominating in the hot spring, overlapping the other thermophiles, especially Archaea;
- and bacterial mesophiles are not random components of this hot spring community and their genomes are replete with genes crucial for hydrothermal and other stress adaptation;

However, the data, obtained by *in silico* analysis of MAGs, does not support these conclusions.

RESPONSE: We thank the reviewer for these comments, and believe that the reviewer appreciated the underlying science of this study.

So far as the lack of conclusive evidence is concerned, it is noteworthy that the holistic elucidation of the microbiome structure and functions for the boiling vent-waters of Trans-Himalayan sulfur-borax hot springs is an ongoing process. We had previously outlined the alpha diversity of the system via analysis of amplified 16S rRNA gene sequences, here we explored the microbiome via deep shotgun metagenomics, and in the very near future we shall communicate the detailed metatranscriptome analyses for this unique microbiome.

Please, find all comments and observations in the attached document

Specifically:

- In the article the authors did not indicate the method by which the functional analysis of the metagenome was performed. As I realized from the manuscript, the metagenome was annotated only as CDSs by searching against the eggNOG database. But this is the first step in *in silico* analysis and it can only reveal general, "raw" things about metabolism of community’s components. The functions revealed in this way only provide directions in which researchers can ‘dig’ further and deeper to understand the nature of what is going on. Relying only on such analysis is very naive and rather superficial.

RESPONSE: We agree that there were certain inadequacies in our metagenomic data mining for certain specialized metabolisms. These shortcomings have now been taken care of by new analyses as suggested by the Reviewer and responded by the authors below.

Moreover, all the details of the bioinformatic procedure that were previously given in the Supplementary Methods, have now been brought to the main text with additional critical details to add to the reliability of the data (please see lines 167-321 of the Track Changes file).

- Secondary metabolites biosynthesis clusters are usually identified by antiSMASH program (doi:10.1038/nrmicro3330) with strict search parameters. Homologues must be found for each identified possibility. We need to realize that we are only talking about secondary metabolites (these are not equal to “antibiotic compounds”), and they can perform many different functions in prokaryote metabolism. In addition, it is necessary for proofing to find genes for all proteins of antibiotic compound biosynthesis, only fragments are not enough. It is also desirable to identify genes encoding transporters, regulating enzymes and so on. In ideal case it is necessary to confirm the assumptions by ‘wet’ experiments or at least references to the literature. And I am not aware of any cases (*in silico* or *in vitro*) of identification of antimicrobial activity in *Aquificota* representatives. The same situation with genes with antibiotic resistance. Usually the ABRicate tool (https://github.com/tseemann/abricate) or ResFinder (doi: 10.1093/jac/dkaa345) and the CARD database (doi:10.1128/AAC.00419-13) are used for such analysis. The analysis in Mondal et al. is based on CDSs predictions only, which is too general and not precise.

RESPONSE: We agree, so have now annotated the assembled metagenome using antiSMASH bacterial version v7.0 with strict detection parameters (Blin et al., 2023) on one hand, and the Resistance Gene Identifier (RGI) v6.0.3, employing Comprehensive Antibiotic Resistance Database (CARD) v3.2.8 (Alcock et al., 2023), on the other. The final results have been upgraded by collating these data with the details of the key genes as well as other associated genes identified for secondary metabolite / antibiotic biosynthesis and resistance by searching against eggNOG database v5.0 using eggNOG-mapper v2.1.9 (please see lines 245-252 of the Track Changes file).

That said, we have now significantly downsized the overall text volume devoted to metagenomic data mining results and corresponding discussions on antibiosis (This included removal of Figure 6). We have also removed the issue altogether from the title, and explicitly mentioned in the few remaining lines of relevant discussion that any activity of the Lotus Pond microflora in this regard is purely potential and subject to actual experimental verification via future studies of culture-dependent microbiology and gene expression analysis (please see lines 59-80, 816-913, 1182-1237, 1270-1275 of the Track Changes file).

- The authors postulated that the *Aquificota* acted as the key primary producers of the ecosystem of Lotus Pond, since genes of rTCA cycle were found in MAGS affiliated to *Hydrogenobacter* close relative. First, the presence of rTCA in *Aquificota* and their role as primary producers in hot spring communities is a long-known fact; second, the genes found in MAGs in this work cannot strictly relate to the rTCA, since they all relate to the TCA or even to ‘exit’ proteins from this pathway. To distinguish TCA from rTCA, one reaction must be found: instead of citrate synthase, a gene for citryle-CoA lyase/ATP-citrate lyase must be present. This is explained in details in the Berg et al., 2011 article referenced by the authors. And a small addition, not relating to the serious inaccuracies of the work, but showing not a full knowledge of the issues discussed there, that nowadays 7 (or even 9, depending on the scientists' opinion) pathways of autotrophic CO_2_ assimilation are already known (DOI: [10.1126/science.aao2410](https://doi.org/10.1126/science.aao2410); DOI: [10.1126/science.aao3407](https://doi.org/10.1126/science.aao3407); doi/10.1073/pnas.1904225116; doi: 10.1038/s41467-020-18906-7), and new pathways have also been identified in the representatives of *Aquificota*.

RESPONSE:

When a new type of ecosystem is explored it is incumbent on the authors to identify its key primary producers in order to generate a comprehensive system level understanding of the habitat. So the objective here is not that something unexpected ought to be discovered, and it is completely another matter that the presence of the pathway or its major players is long known for other comparator ecosystems.

We agree with the second point, so have now removed the asides and specifically highlighted the presence of the signature genes of (1) the rTCA cycle, (ii) the reductive pentose phosphate cycle, (iii) the 3-hydroxypropionate bi-cycle, (iv) the reductive acetyl Co-A pathway, (v) the dicarboxylate/4-hydroxybutyrate cycle and (vi) the 3-hydroxypropionate/4-hydroxybutyrate cycle, both in the metagenome and the MAGs. Examples of such key genes include the one encoding for ATP-citrate lyase and citryl-CoA lyase that differentiate the rTCA and modified rTCA cycles respectively from the TCA cycle for the overhauled data and discussion on the key genes of all the pathway detected (please see lines 614-746 of the Track Changes file). Notably, these key genes were shown in our previous Figure 5 but were not underscored in the text or the legend, so we have upgraded Figure 5 by highlighting the key enzymatic steps of all the major autotrophic pathways. Furthermore, we have discussed the lately reported and not so common pathways, namely (vii) the reverse oxidative TCA (roTCA) cycle, (viii) the transaldolase variant of the Calvin cycle mediated by Form III RubisCO, and (ix) (viii) the reductive glycine pathway, in their proper perspectives (please see lines 606-613 of the Track Changes file). As pointed out by the Reviewer,

Nunoura et al. Science, 2018; 359:559-563;

Mall et al. Science, 2018; 359:563-567;

Sánchez-Andrea et al. Nat Commun, 2020; 11:5090; and

Frolovet al. 2019; Proc. Natl Acad Sci USA, 116:18638-18646

have been cited in this regard.

- The work focuses very much on the diversity, relative abundance and capabilities of mesophilic prokaryotes in analyzed extremely hot spring (85°C). However, estimating their representation in the community by CDSs gave an inadequate result, because:1) it doesn't mention what method was used to determine that; 2)the genome sizes of thermophilic prokaryotes belonging to such truly deep-phylogenetic lineages, such as *Aquificota*, *Thermotogota*, *Thermoproteota* etc., are 2-3 times smaller than those of mesophilic bacteria. Accordingly, the number of genes in genomes of thermophiles is much less. Herewith the 16S gene metagenomic analysis reflected the usual pattern for such conditions quite properly, where *Aquificota* vastly dominated over the others. Moreover, the stained cells counting experiment (by the way, it was completely absent in the Discussion) revealed approximately 63,5% (5,4/8,5*100%) of metabolically viable cells, that could be all truly thermophiles. No evidence was given to prove that mesophiles could contribute to living cells quantity. Real time PCR, or FISH with specific probes engineering to mesophilic taxons, or cultivation of water/filter sample in mesophilic conditions and enriching any mesophilic culture (with strict controls, of course) might help in this situation. And the additional research on 16S amplicons with different primers only confuses the reader. It is not very clear to me why it was necessary to carry out such an analysis if it has already been done in previous works by the same group of authors. This article is not a methodological work, so why authors devoted so much time and efforts to comparison them?

RESPONSE: We thank the Reviewer for the minute examination of our work. Indeed, under the existing state of knowledge on the microbial ecology of the poorly studied Trans-Himalayan sulfur-borax hot springs, the central objective of this paper is to holistically elucidate the microbiome structure and functions of the vent-water of one representative spring of this geothermal area.

We agree that in the main text of the previous manuscript an inadequacy was apparent with regard to the methods used (especially by means of CDS annotation) for estimating the representation of the various taxa in the community. This perception emanated primarily due to the fact that in the previous manuscript, all the details of the bioinformatic procedures concerned with metagenomics were given in the Supplementary Methods. We have now brought all those critical information to the main text to add to the reader’s satisfaction as well as the study's reliability (please see lines 229-252 of the Track Changes file).

Thus, in the revised Article File,

we have now elaborated the previous Methods section called “Metagenomics” under three new sections titled as

- Metagenome extraction and sequencing
- Assembly and annotation of the metagenomic sequence
- Direct annotation of metagenomic reads corresponding to rRNA gene sequences,

besides significantly substantiating the Methods sections

- Construction and taxonomic characterization of metagenome-assembled genomes (MAGs)
- PCR-amplified 16S rRNA gene sequence analysis (for the upgraded methods pertaining to MAGs and OTUs please see lines 197-321 of the Track Changes file).

We also agree that the genome sizes of thermophilic prokaryotes belonging to such the deep-phylogenetic lineages, such as *Aquificota*, *Thermotogota*, *Thermoproteota* etc., are 2-3 times smaller than those of mesophilic bacteria. Hence, the numbers of genes in genomes of those thermophiles are much less. But this does not falsify the conclusion that most part of the genetic diversity present in the microbiome is contributed by phylogenetic relatives of mesophilic taxa, even though they are not numerally predominant (as subsequently evident from the direct annotation of metagenomic reads corresponding to rRNA gene sequences), and also perhaps not alive or metabolically active in large numbers.

It is unequivocally true that in the current paper we have given no evidence to prove that mesophiles at all contributed to the live microflora of the Trans-Himalayan sulfur-borax hot spring explored. In that context it is noteworthy that the holistic elucidation of the microbiome structure and functions for the boiling vent-waters of Trans-Himalayan sulfur-borax hot springs is an ongoing process and we are proceeding in a step-wise manner. We had previously outlined the alpha diversity of the system via analysis of amplified 16S rRNA gene sequences (Roy et al. Front Microbiol. 2016, 7:412; Roy et al. J Earth Syst Sci. 2020, 129:157; Mondal et al. J Earth Syst Sci. 2022, 131:12), then we isolated a few strains that were phylogenetically related to mesophilic bacteria (Roy et al. Front Microbiol. 2016, 7:412; Mondal et al. Microbiol Spectr. 2022, 10:e01606-22); here we explored the microbiome via deep shotgun metagenomics, and in the very near future we shall communicate the detailed metatranscriptome analyses for some of these unique microbiomes alongside more results pertaining to the cultivation of water samples under mesophilic conditions and enrichment and isolation of mesophilic cultures.

The following points would remove the Reviewer’s confusion in relation to the apparently-redundant data provided in this paper on the basis of 16S amplicon sequencing. It is necessary to carry out such analyses because one of the key objectives of the current work is to know whether the Lotus Pond vent-water microbiome remained consistent over time. Since 16S amplicon based works had been done previously to reveal the alpha diversity of Lotus Pond’s vent-water, a robust assessment of the temporal stability of the microbial diversity could only be achieved if comparisons were done between equivalent data types, i.e. old 16S ampliseq data versus new 16S ampliseq data, and a new WGS versus old 16S ampliseq comparison would not have been sufficient (or even appropriate) in this regard.

Furthermore, a comparison between the WGS data and the 16S ampliseq data was necessary because several discrepancies and concurrence were indeed there in the two categories of results. Had we not discussed these issues in their proper perspectives, the reliability and credibility of the whole gamut of findings would have been compromised.

- Presence of mesophilic DNA in the sample from the hot spring with 85°C can be explained by the following factors: 1) Dirty sampling; 2) Low concentration or low quality of the total DNA (the authors did not provide any evidence to the contrary, and the low cell counts in the study – 10^4^ cells/ml - may indicate at least a low amount of DNA in the analysis); 3) Mesophiles may be transferred accidentally to hot spring from cold habitats nearby with precipitation, plant fall, river, etc. In the case of Lotus Pond, a geyser, each time discharging to the surface, may capture nearby cold areas and flush mesophiles into itself with a strong stream of water. But in any case mesophiles cannot be considered as true components of the hot spring community. This is supported by the facts that there are no pure cultures of mesophiles capable of growing (not surviving for a short period of time) at 85°C, and I don’t know any ecological articles devoted to microbial communities of other hydrothermal systems with such extreme temperatures (like in YNP, Iceland, Russia, Japan hot springs) where it was proven that mesophiles were there not by a chance. In this research, the authors providedreferences only by their group’s works and only in this region. But this is a little confusing.

RESPONSE: The Reviewer’s apprehension regarding “dirty sampling” has plausibly emanated from the absence of a routine description of the sampling procedure in the previous version of the manuscript. The details of the meticulous care that was taken during sample collection to avoid contamination were all described at length in our previous publications dealing with this system (Roy et al., 2016, 2020a, 2020b; Mondal et al., 2022). We therefore thought that citing those papers would be sufficient here and reiteration of the same old points was unwise and, hence unnecessary. However, we have now inserted some of the critical points to the text pertaining to “sampling” so as to add to the reader’s satisfaction as well as the study's reliability (please see lines 167-179 of the Track Changes file).

So far as DNA-level contamination is concerned, we apologize for forgetting to include in the previous manuscript the negative findings of the experiments which we had carried out to detect potential traces of contaminating microial DNA (which is often referred to as the “kitome”) in the Lotus Pond metagenomic sequence. All kitome-related procedures as well as the negative results have now been incorporated to the revised manuscript (please see lines 214-222, 311-314 of the Track Changes file).

Indeed, mesophiles get transferred stochastically to these hot springs from adjoining cold habitats. For Lotus Pond, this geodynamic phenomenon has been discussed at length in our previous paper Roy et al. 2020 (Geomicrobial dynamics of Trans-Himalayan sulfur–borax spring system reveals mesophilic bacteria’s resilience to high heat J. Earth Syst. Sci. 129:157). The phenomenon of mesophilic microorganisms getting introduced to hot spring systems has been reported from other parts of the world too; for instance, systemic infiltration of mesophiles from surface and sub-surface sources of cold water and meteoric fluids have been observed for hot springs of New Zealand and Yellowstone National Park, USA (Power et al. 2018 Microbial biogeography of 925 geothermal springs in New Zealand; Nat. Commun. 9: 2876; Colman et al. 2019 Mixing of meteoric and geothermal fluids support hyperdiverse chemosynthetic hydrothermal communities; Nat. Commun. 10: 681). We had cited these corroborative publications in our 2020 paper in J. Earth Syst. Sci. 129:157, but thought that these aspects were not so relevant in the context of the present paper. Anyway, to assuage the concerns of the Reviewer we have now elaborated on the topic of mixing of meteoric water with geothermal fluids, and in doing so included the above citation in the revised manuscript (please see lines 108-126 of the Track Changes file).

We agree that there is still no conclusive evidence to prove that mesophiles contribute to the live microflora of the Trans-Himalayan sulfur-borax hot spring explored. In that context it is noteworthy that the holistic elucidation of microbiome structure and functions for the boiling vent-waters of Trans-Himalayan sulfur-borax hot springs is an ongoing process and we are proceeding in a step-wise manner. We had previously outlined the alpha diversity of the system via analysis of amplified 16S rRNA gene sequences (Roy et al. Front Microbiol. 2016, 7:412; Roy et al. J Earth Syst Sci. 2020, 129:157; Mondal et al. J Earth Syst Sci. 2022, 131:12), then we isolated a few strains that were phylogenetically related to mesophilic bacteria (Roy et al. Front Microbiol. 2016, 7:412; Mondal et al. Microbiol Spectr. 2022, 10:e01606-22); here we explored the microbiome via deep shotgun metagenomics, and in the very near future we shall communicate the detailed metatranscriptome analyses for some of these unique microbiomes alongside more results pertaining to the cultivation of water samples under mesophilic conditions and enrichment and isolation of mesophilic cultures.

I think it's an interesting idea that *Aquificota* may contain in their genomes a number of genes for the synthesis of antibiotic compounds, which could inhibit the growth of other thermophiles and help them to dominate in almost all hydrothermal areas worldwide. And if the authors decide to prove this hypothesis, this study requires additional experiments, particularly proper *in silico* analysis of MAGs and/or genomes and *in vitro* antibiotic activities of pure cultures of *Aquificota* at least. And in this case it will be a truly novel bright work. But in the meantime the work is too surface level, despite the time and efforts put into writing it. The results of the analysis presented in the article do not support the title or what is put in the abstract.

RESPONSE: We agree so have now significantly downsized the metagenomic data mining results and corresponding discussions on antibiosis (This included removal of Figure 6). We have also removed the issue altogether from the title, and explicitly mentioned in the few remaining lines of relevant discussion that any activity of the Lotus Pond microflora in this regard is purely potential and subject to actual experimental verification via future studies of culture-dependent microbiology and gene expression analysis (please see lines 59-80, 816-913, 1182-1237, 1270-1275 of the Track Changes file).

**Response to the comments of Reviewer 4**

Interesting and very well written manuscript. I have only one reservation - you analyzed just the metagenome, so, the conclusions of the article and partially even the title of the article are not based on experimental data. The presence of a large number of "resistance" genes is not an evidence that these genes are actually expressed. For many bacterial taxa, it is well documented that these genes or gene clusters are not actually expressed.

Without additional transcriptomic data, in my opinion, the title of the article is inappropriate

RESPONSE: We agree so have now significantly downsized the metagenomic data mining results and corresponding discussions on antibiosis (This included removal of Figure 6). We have also removed the issue altogether from the title, and explicitly mentioned in the few remaining lines of relevant discussion that any activity of the Lotus Pond microflora in this regard is purely potential and subject to actual experimental verification via future studies of culture-dependent microbiology and gene expression analysis (please see lines 59-80, 816-913, 1182-1237, 1270-1275 of the Track Changes file).
